# Flagellins as Vaccine Adjuvants and Cancer Immunotherapy: Recent Advances and Future Prospects

**DOI:** 10.1111/imm.70001

**Published:** 2025-06-10

**Authors:** Asma Talukder, Md. Mijanur Rahman, Md. Sifat Rahi, Dean L. Pountney, Ming Q. Wei

**Affiliations:** ^1^ School of Pharmacy and Medical Sciences Griffith University Gold Coast Queensland Australia

**Keywords:** adjuvants, cancer, flagellin, immunotherapy, infectious diseases, vaccine

## Abstract

Flagellin, an essential structural protein of bacterial flagella, has emerged as a potent modulator of both specific and nonspecific immunity, demonstrating significant potential as a vaccine adjuvant and carrier. By inducing the release of pro‐inflammatory cytokines like IL‐1β, TNF‐α, IL‐6, IL‐8, and IL‐12, flagellin activates the innate immune system, enhancing antigen‐specific adaptive immune responses mediated by tumour‐specific type 1 helper T cells and cytotoxic T cells, thus positioning it as a valuable adjuvant or complementary therapy for various cancers and infectious diseases. This review explores recent strategies, innovations, and clinical applications of flagellin‐based immunotherapies, particularly in the context of infectious diseases and cancers. Flagellin from *Salmonella typhimurium* has been extensively studied as a vaccine adjuvant for diseases like HIV, influenza, dengue, West Nile virus, poultry cholera, and bursal diseases and shows promise in treating lung metastasis, melanoma, colon, and prostate cancers. It has also proven effective against multidrug‐resistant bacteria, including *Pseudomonas aeruginosa* and *S. typhimurium* . Notably, *S. typhimurium* flagellin‐based vaccines for influenza have progressed to clinical trials. Additionally, flagellins from *S. typhi* , *S. enteritidis* , *P. aeruginosa* , and *Escherichia coli* are being evaluated as vaccine candidates for plague, malaria, and infections caused by *P. aeruginosa* and *E. coli* . In cancer therapy, flagellin‐based treatments, especially when combined with tumour antigens, have exhibited the ability to enhance anti‐tumour immunity and improve patient outcomes. Other flagellin‐based vaccines derived from *S. Dublin*, *S. munchen*, and *Vibrio vulnificus* have been employed in the treatment of prostate, lung, liver, breast, cervical, and colorectal cancers, as well as lymphoma, melanoma, and radiation‐induced mucositis. Mobilan, a recombinant non‐replicating adenovirus vector expressing *Salmonella* flagellin, is currently in a phase Ib clinical trial for prostate cancer. Overall, bacterial flagellin treatments are generally safe, well‐tolerated, and associated with minimal side effects, making them a promising option for managing infectious diseases and cancers.

## Graphical abstract

The figure depicts the role of flagellin in immune cells. Flagellin interacts with TLR5, resulting in the formation of homodimers and the recruitment of MyD88, which activates the IκB and MAPK signalling pathways. This results in the activation of NF‐κB and AP‐1, producing TNF‐α, IL‐6, IL‐8, and IL‐12. Flagellin interacts with TLR5/TLR4 heterodimers, activating the IRF3 pathway and leading to IFN‐β production, activating the JAK–STAT pathway and enhancing NO synthesis. Intracellular flagellin is recognised by NAIP5, leading to the formation of an inflammasome complex with NLRC4, which activates caspase‐1 and processes IL‐1β and IL‐18, essential for host defence.

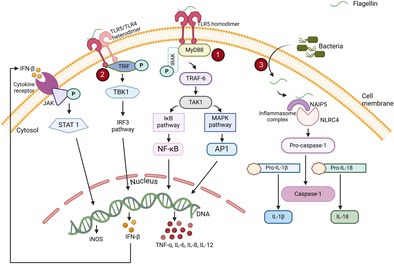

**Abbreviations**
AI: artificial intelligence
AIV: avian influenza virus
AP‐1: activator protein‐1
APC: antigen‐presenting cell
BALF: bronchoalveolar lavage fluids
CpG‐ODNs: cytosine‐phosphate‐guanine oligodeoxynucleotides
CRP: C‐reactive protein
CTCAE: common terminology criteria for adverse events
CTL: cytotoxic T lymphocyte
CTLA‐4: cytotoxic T lymphocyte antigen‐4
DC: dendritic cell
DENV: dengue virus
ENA‐78: epithelial neutrophil activating peptide‐78
FaeG: primary subunit of F4 fimbriae
FasL: Fas ligand
FlaB:
*Vibrio vulnificus* flagellin
FMDV: foot and mouth disease virus
fPlpE:
*Pasteurella multocida* lipoprotein E
FRNT_50_
: 50% focus reduction neutralisation tests
G‐CSF: granulocyte colony‐stimulating factor
gLN: genital lymph node
GMT: geometric mean titres
GRO‐α: growth regulated oncogene‐α
HA: hemagglutinin
HAI: hemagglutination inhibition
HIV: human immunodeficiency virus
HPV: human papillomavirus
IBD: infectious bursal disease
IBDV: infectious bursal disease virus
IFN: interferon
Ig: immunoglobulin
IkB: inhibitor of kappa B
IL: interleukin
iNOS: inducible nitric oxide synthase
IPAF: ICE protease activating factor
IRAK: interleukin‐1 receptor‐associated kinase
IRF3: transcription factor interferon regulatory factor 3
IU: international units
IVAG: intravaginal administration
JAK: Janus kinase
LFPS: lipid‐A free lipopolysaccharide
LPS: lipopolysaccharide
MAPK: mitogen‐activated protein kinase
MDC: macrophage derived chemokine
MDR: multidrug resistant
MHC: major histocompatibility complex
MIP‐3α: macrophage inflammatory protein‐3α
mL: millilitre
MSP1_19_
: 19 kDa C‐terminal region of *Plasmodium vivax* merozoite surface protein‐1
MUC1: tumour‐associated antigen mucin 1
MyD88: myeloid differentiation primary response 88
NAIP: NLR family apoptosis inhibitory protein
NF‐κB: nuclear factor kappa‐light‐chain‐enhancer of activated B cells
ng: nanogram
NHP: non‐human primate
NIH: National Institutes of Health
NK: natural killer cells
NLR: NOD‐like receptor
NLRC4: NOD‐like receptor (NLR) family caspase activation and recruitment domain‐containing protein 4
NO: nitric oxide
NOD: nucleotide‐binding oligomerization domain containing protein
PAMP: pathogen‐associated molecular pattern
PBMC: peripheral blood mononuclear cell
PD: programmed cell death protein
PD‐L: programmed cell death ligand
PRR: pattern recognition receptor
RPS: relative percentage survival
SDS‐ PAGE: sodium dodecyl sulphate‐polyacrylamide gel electrophoresis
SLAM: signalling lymphocytic activation molecule
STAT: signal transducer and activator of transcription
STF:
*Salmonella typhimurium* flagellin
STF2: STF phase 2
STF2Δ: modified version of STF2
STFΔ: truncated version of STF
T3SS: type III secretion system
TAK: transforming growth factor‐beta‐activated kinase
TBK: TANK‐binding‐kinase
TDV: tetravalent dengue vaccines
Th: helper T cells
TLR: toll‐like receptor
TLR5M: membrane‐bound TLR5
TLR5S: soluble form of TLR5
TME: tumour microenvironment
TNF: tumour necrosis factor
TRAF: TNF receptor‐associated factor
TRIF: TIR domain‐containing adapter inducing interferon‐β
VLP: virus‐like particles
μg: microgram

## 1 Introduction

The flagellum is the organelle responsible for the movement of bacterial cells. The flagellar activity is linked to the chemotaxis system that detects ambient chemical and physical stimuli and coordinates cellular movement for bacterial development and survival [1, 2, 3]. It is an elongated filamentous structure on the surface of bacteria, consisting of three components: the basal body, the hook, and a helical hollow filament, and thousands of copies of the protein named flagellin make up the filament [4, 5]. The entire flagellin structure is categorised into four domains, designated D0, D1, D2, and D3. The domains are organised from the inside to the outside of the filament. The N‐terminal chain begins at D0, traverses D1 and D2, arrives at D3, and, after that, retraces its path via D2 and D1; the C‐terminal chain concludes at D0. Consequently, the overall morphology of flagellin, when seen perpendicular to the filamentous axis, resembles an uppercase Greek gamma (Γ) [6, 7].

Flagellin functions as a pathogen‐associated molecular pattern (PAMP) recognised by the host innate immune system's pattern recognition receptors (PRRs), namely toll‐like receptor (TLR) 5 on the cell surface and the NOD‐like receptor (NLR) family caspase activation and recruitment domain‐containing protein 4 (NLRC4) in the cytoplasm [8, 9, 10, 11]. The activation of PRRs initiates innate immune signalling pathways, leading to the rapid release of pro‐inflammatory cytokines and the activation of antigen‐presenting cells (APCs), which subsequently facilitate the onset of antigen‐specific adaptive immunological responses [10]. PRRs are primarily produced by innate immune cells such as macrophages, monocytes, dendritic cells (DCs), and neutrophils, and are crucial in connecting innate and adaptive immunity [12, 13, 14].

Since current therapeutic and preventive approaches are frequently constrained by insufficient efficacy, resistance, or immune evasion mechanisms, infectious diseases and cancer continue to pose serious threats to global health. As a result, improving host immune responses is now a primary goal in the creation of next‐generation therapies. In this sense, immunostimulants are essential to the development of contemporary vaccines, and an enormous amount of research is being done to create new substances that can boost immune responses against tumour cells as well as microbial infections [10, 12, 14, 15, 16, 17] Flagellin has been used as an adjuvant in vaccines and cancer immunotherapy [18, 19, 20] due to its ability to activate both TLR5 and NLRC4 pathways, which primarily stimulate innate immunity, therefore eliciting humoral immune responses [14, 15, 21]. These events include the synthesis of pro‐inflammatory cytokines and chemokines by immune cells, the activation of APCs, the maturation of DCs, their passage to lymph nodes, and the immediate activation of T and B lymphocytes to produce antibodies [14, 22, 23]. By boosting antibody production, flagellin plays an important role in creating long‐lasting immune memory. This broad activation process brings two significant biological advantages: it helps to prevent infections and tumours by establishing robust immune surveillance, and aids in treatment by activating immune mechanisms that can identify and eliminate already infected or cancerous cells [14, 24, 25].

While flagellin‐based vaccines show great potential for boosting the immune system and many have successfully progressed to clinical studies in humans for infectious diseases and cancers [14, 26, 27, 28, 29, 30, 31, 32], their clinical development has faced several obstacles. Conventional adjuvants like alum, cytosine‐phosphate‐guanine oligodeoxynucleotides (CpG‐ODNs), and MF59 have long been used in approved vaccines, supported by a wealth of data on their safety and effectiveness [16, 33, 34]. Alum, one of the first adjuvants to be approved for use in humans, is particularly known for enhancing humoral immunity by triggering type 2 helper T (Th) cell responses and is esteemed for its stability, minimal risk of causing adverse reactions, and low production cost. However, it often fails to elicit robust cellular immunity, hence limiting its efficacy against intracellular infections [35, 36, 37]. CpG‐ODNs, which mimic bacterial DNA and specifically target TLR9, elicit strong Th1 responses and boost the activity of cytotoxic T lymphocytes (CTLs), making them particularly useful in vaccines to fight intracellular pathogens and tumours. While CpG‐ODNs have proven effective, they can also pose some challenges, such as stability concerns and the potential for overstimulating immune cells in certain groups [38, 39, 40]. MF59 is an oil‐in‐water emulsion derived from squalene that offers a more balanced profile. It enhances both humoral and cellular immune responses by improving antigen absorption and increasing the recruitment and activation of APCs at the injection site. The primary strength of MF59 is its capacity to augment responses to weak antigens, making it quite versatile [41, 42].

Nonetheless, flagellin is particularly notable for its dual role as an adjuvant and an immunogen, providing the necessary signals for immune activation. It is especially promising for mucosal vaccines and cancer immunotherapy, as it can trigger both systemic and mucosal immunity, accompanying responses from CTLs [10, 22, 43]. However, these benefits are counterbalanced by its high immunogenicity, which can result in mild to moderate side effects such as fatigue, headache, and muscle aches [29, 44, 45], along with prolonged systemic inflammation, especially attributed to the increased activation of TLR5 in both lymphoid and non‐lymphoid tissues. When TLR5 is overactivated, it can lead to an excessive production of interleukin (IL)‐6, which then triggers the release of C‐reactive protein (CRP) in the blood. This may generate inflammatory consequences, including sepsis‐like symptoms or autoimmune exacerbations in susceptible people [28, 31, 46].

Moreover, while alum and MF59 have established formulation platforms, flagellin‐based formulations often struggle with issues like protein stability, aggregation, and scaling up for manufacturing. The move into late‐phase clinical trials has been largely held back by safety concerns and the necessity to improve delivery methods and dosage guidelines. Additionally, the risk of cytokine storms and the development of anti‐flagellin antibodies pose significant challenges for its long‐term clinical application and commercialisation [14, 45, 47].

This review highlights recent research advancements on the role of bacterial flagellin in modulating immune responses as an immunostimulant for vaccine development in cancer immunotherapy and infectious diseases, supported by promising clinical and preclinical evidence demonstrating enhanced vaccine efficacy and therapeutic outcomes.

## 2 Mode of Action of Flagellin as Immunostimulant

Flagellin, unequivocally the key component of flagella, serves as a PAMP that interacts with various receptors, triggering complex signalling pathways to activate both innate and adaptive immune responses [10, 15]. The broad adjuvant effect of flagellin is ascribed to the presence of TLR5 by several immune cell types such as myeloid DCs, monocytes, macrophages, T cells, Langerhans cells, natural killer (NK) cells, epithelial cells, and polymorphonuclear neutrophils [48, 49, 50, 51]. Extracellular flagellin–TLR5 interaction activates several essential genes via myeloid differentiation primary response 88 (MyD88)‐dependent or MyD88‐independent pathways [52, 53]. Subsequently, the relevant cells are stimulated to generate various cytokines and co‐stimulatory molecules, including the synthesis of tumour necrosis factor (TNF)‐α and the expression of signalling lymphocytic activation molecule (SLAM), a hallmark of activation from monocytes [54]; the production of IL‐8, TNF‐α, interferon (IFN)‐γ and nitric oxide (NO) from epithelial cells [55, 56], the secretion of IL‐6, IL‐12 from DCs [57, 58]; the generation of IL‐6, IL‐8, IL‐12, TNF‐α, and IL‐1β from macrophages [59, 60]. Flagellin–TLR5 signalling proceeds through a MyD88‐dependent adaptor protein which is pivotal for downstream signalling and facilitates the formation of a TLR5 homodimer that transmits the signal to the inhibitor of kappa B (IkB) and mitogen‐activated protein kinase (MAPK) cascades via interleukin‐1 receptor‐associated kinase (IRAK), TNF receptor‐associated factor (TRAF) 6, and transforming growth factor‐beta‐activated kinase (TAK) 1. The IkB and MAPK pathways cause the activation of the transcription factors nuclear factor kappa‐light‐chain‐enhancer of activated B cells (NF‐κB) and activator protein‐1 (AP‐1), respectively. The two mentioned transcription factors lead to the activation of several pro‐inflammatory cytokine genes, including TNF‐α, IL‐6, IL‐8, and IL‐12, which are engaged in both innate and adaptive immunity [61, 62].

In contrast, flagellin also engages TLR5/TLR4 heterodimers that are independent of MyD88, leading to the activation of the TIR domain‐containing adapter inducing interferon‐β (TRIF) adaptor protein. This process stimulates the interferon regulatory factor 3 (IRF3) pathway through TANK‐binding‐kinase (TBK) 1, resulting in the transcription of the IFN‐β gene. The production of IFN‐β triggers the janus kinase‐signal transducer and activator of transcription (JAK–STAT) signalling cascade, promoting the synthesis of NO by stimulating inducible nitric oxide synthase (iNOS) [14, 53].

**FIGURE 1:**
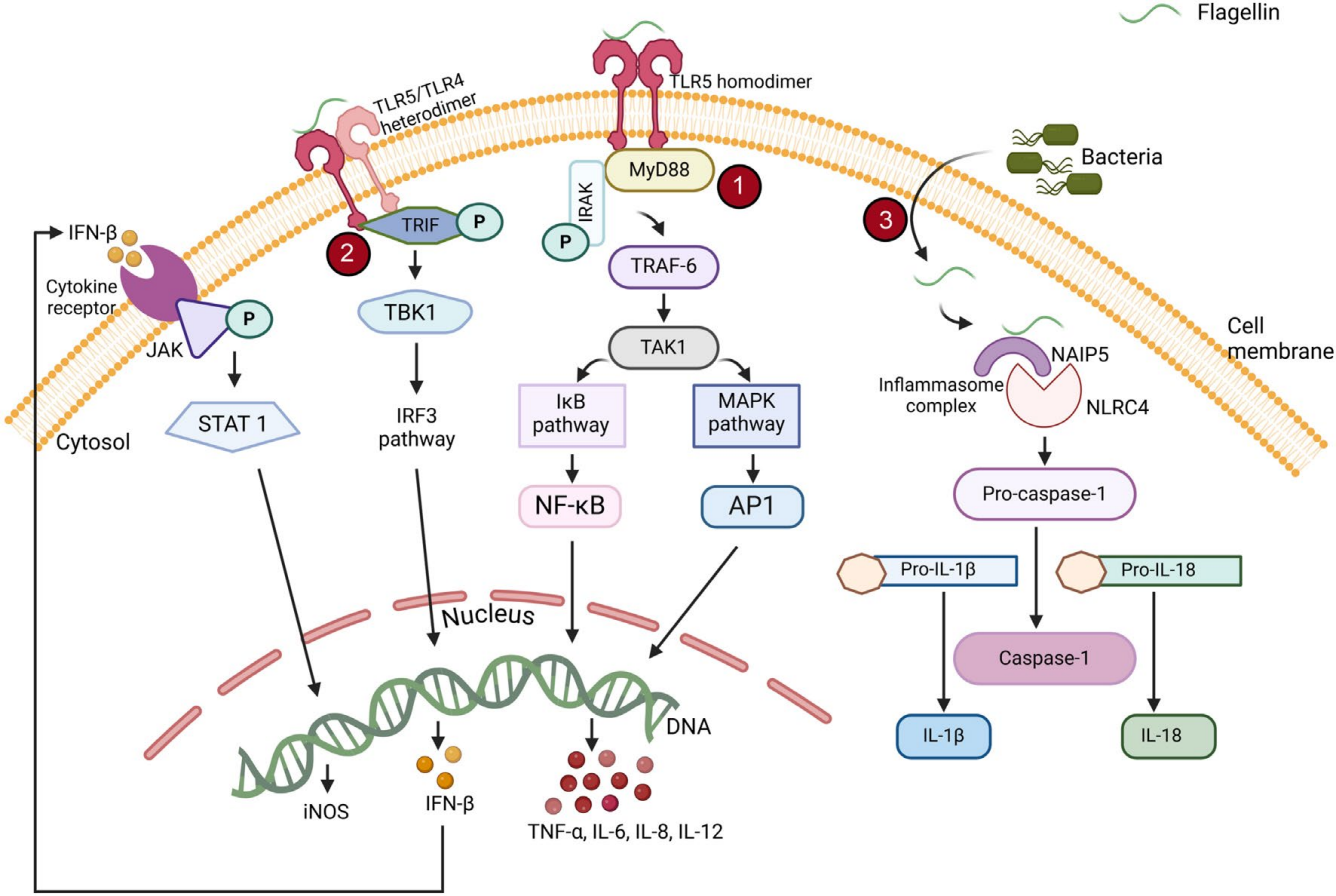
Mode of action of flagellin. The binding of flagellin to TLR5 on the extracellular surface triggers the formation of TLR5 homodimers. This interaction brings in the adaptor protein MyD88, which activates two key signalling pathways: The IkB and MAPK pathways. These pathways lead to the activation of transcription factors NF‐κB and AP‐1, respectively. As a result, cytokines such as TNF‐α, IL‐6, IL‐8, and IL‐12 are produced, all of which play a role in type 1 immunity (1). Besides homodimer signalling, flagellin can also interact with TLR5/TLR4 heterodimers, activating the IRF3 pathway that results in the transcription of the IFN‐β gene (2a). The production of IFN‐β then activates the JAK–STAT signalling pathway, which enhances the synthesis of NO (2b). Additionally, flagellin can be introduced into the host cell cytoplasm via bacterial type III secretion systems. Once inside, intracellular flagellin is detected by NAIP5, leading to the formation of an inflammasome complex with NLRC4. This inflammasome activates caspase‐1 by converting it from its inactive form, procaspase‐1, into its active state. The active caspase‐1 then processes the precursors of IL‐1β (pro‐IL‐1β) and IL‐18 (pro‐IL‐18) into their active forms, IL‐1β and IL‐18, which are essential for the host defence mechanism (3).

However, the delivery of flagellin into the cytoplasm of host cells through the bacterial type III secretion system (T3SS) triggers a strong immune response mediated by NLRC4, which is also referred to as ICE protease activating factor (IPAF). Many Gram‐negative bacteria, such as *Salmonella*, utilise the T3SS, a specialised protein complex, to directly inject effector proteins like flagellin into the host cell cytoplasm [11, 63]. Once flagellin is inside the cytoplasm, it is recognised by NLR family apoptosis inhibitory protein (NAIP) 5, a cytoplasmic innate immune sensor [64]. The interaction between flagellin and NAIP5 leads to the formation of an inflammasome complex with NLRC4, which is crucial for converting pro‐caspase‐1 into its active form, caspase‐1. This activated caspase‐1 then processes pro‐IL‐1β and pro‐IL‐18 into their active forms, IL‐1β and IL‐18, which are vital pro‐inflammatory cytokines necessary for the host's defence [65, 66, 67]. The conserved C‐ and N‐terminal domains of flagellin are essential for its ability to bind to TLRs, while its recognition by NLRC4 relies on specific residues in the C‐terminal region. Mutations in critical leucine residues within the C‐terminal domain hinder the formation of the NAIP5/NLRC4 inflammasome, highlighting the importance of structural specificity for flagellin‐induced innate immune activation [68, 69] (Figure 1).

McEwen, Levi [70] first delineated the adjuvant properties of flagellin. When administered with vaccine antigens, flagellin induces robust systemic and mucosal adaptive immune responses to both flagellin and the accompanying antigens [21, 71]. For vaccine preparation, flagellin can be integrated with foreign antigens via various methods to produce live attenuated vaccines, recombinant protein‐based conjugate vaccines, and combinations for co‐administration. These substances enhance vaccine effectiveness to a particular extent [15, 72]. Adjuvants may enhance the immune response to antigens via several methods by augmenting the circulation of antigens in the bloodstream, boosting the proliferation of APCs, activating macrophages and lymphocytes, and improving the synthesis of cytokines [73, 74].

Adjuvants generally work by creating a depot at the injection site, which allows for a slow and steady release of antigens over a period of 2–3 weeks. This gradual release improves the absorption and presentation of antigens, recruiting immune cells by causing localised inflammation [36, 75]. Additionally, adjuvants affect the expression of genes that produce chemokines like CCL2, CCL3, and CCL5, as well as cytokines such as TNF‐α, IL‐6, IL‐12, and IL‐1β [16, 75, 76]. This environment rich in cytokines promotes immune activation, helping to restore tumour‐specific cytotoxic T cells so they can recognise and respond to tumour antigens [10, 77].

APCs, especially DCs, play a vital role in starting immune responses against tumour antigens. DCs connect innate and adaptive immunity by internalising antigens and presenting them on major histocompatibility complex (MHC) molecules [78]. Immature DCs are particularly good at taking up antigens through phagocytosis and micropinocytosis. In the tumour microenvironment (TME), stimulating immature DCs with TLR ligands can temporarily boost antigen‐specific micropinocytosis [79], which may enhance antigen acquisition when combined with TLR ligand‐based adjuvants. After they take up antigens, DCs increase the expression of MHC I, MHC II, and co‐stimulatory molecules while decreasing their ability to capture more antigens. These antigen‐loaded DCs then move to draining lymph nodes, which are the main sites for T cell priming [80].

**FIGURE 2:**
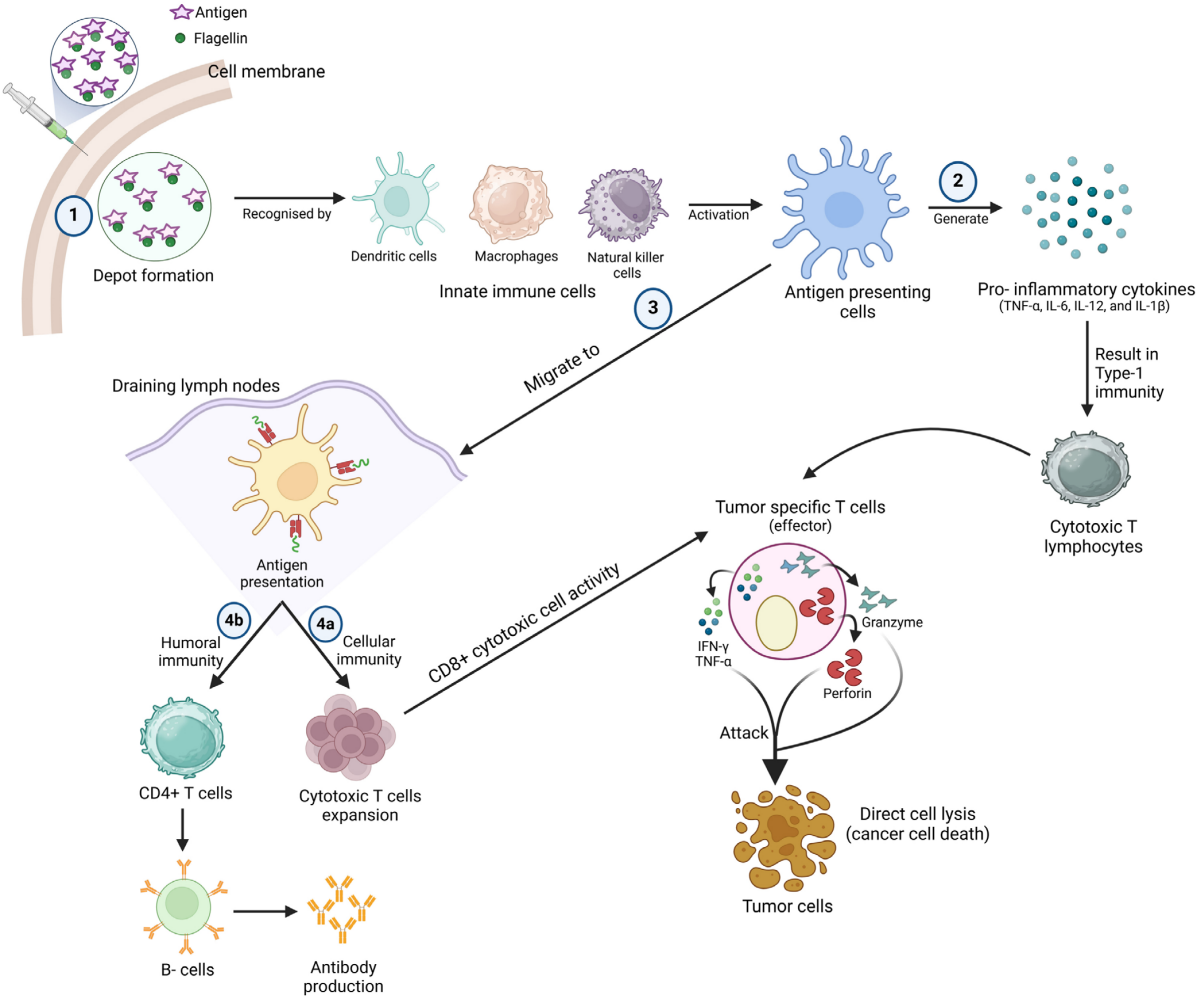
Mechanism of action of flagellin adjuvant‐based vaccine. Injection of flagellin adjuvant‐ based vaccines establishes a depot at the injection site, which allows for the gradual release of antigens, improving their uptake and presentation by APCs (1). This mechanism draws immune cells like DCs, macrophages, and NK cells, triggers localised inflammation and activates these cells to function as APCs. Once activated, APCs release cytokines such as TNF‐α, IL‐6, IL‐12, IL‐1β, and various chemokines linked to immune responses. This surge of cytokines fosters type 1 immunity, enhancing the functions of tumour‐specific CTLs that identify cancer antigens and destroy cancer cells by releasing cytotoxic substances like granzymes, perforins, TNF‐α, and IFN‐γ (2). Furthermore, activated APCs, especially DCs, travel to draining lymph nodes, where they present antigens to CD4^+^ and CD8^+^ T cells (3). Presenting antigens to CD8^+^ T cells stimulate cellular immunity, leading to the proliferation of CTLs and the cytotoxic actions of CD8^+^ T cells, which ultimately result in the elimination of cancer cells (4a). On the other hand, presenting antigens to CD4^+^ T cells trigger humoral immunity by activating B cells, which then produce tumour‐specific antibodies (4b).

In the lymph nodes, mature DCs present processed antigen fragments on MHC I and MHC II molecules to naive CD8^+^ and CD4^+^ T cells, respectively [81]. When CD8^+^ T cells are activated through their antigen receptors, they proliferate and differentiate into effector CTLs. These CTLs then infiltrate the tumour core and work to eliminate tumour cells. The quantity of CTLs in the TME is a crucial prognostic indicator of malignancy. CTLs identify tumour cells exhibiting certain antigens and eliminate target cells in various ways. Initially, CTLs may eliminate cancer cells by synthesising and secreting cytotoxic molecules, including perforin and granzymes. Additionally, CTLs facilitate the death of target cells via Fas ligand (FasL)‐mediated interactions [82]. Moreover, the secretion of IFN‐γ and TNF‐α by CTLs elicits the cytotoxicity of tumour cells [83]. IFN‐γ generated by CTLs facilitates their further differentiation into effector CTLs [84]. Beyond the antigen‐specific CTL responses, DCs also produce IL‐12 and IFN‐γ to enhance the expression of co‐stimulatory signals that are essential for strong T cell activation [85]. The interaction between tumour‐specific T cells and the MHC I peptide complex, along with co‐stimulatory molecules, drives their differentiation into effector and memory T cells. These tumour‐specific effector T cells proliferate and migrate to the TME, where they exert cytotoxic effects and release effector cytokines to help clear the tumour [86]. At the same time, activated B cells differentiate into antibody‐secreting cells that target tumour‐specific antigens, thereby contributing to the humoral aspect of antitumour immunity [87] (Figure 2).

## 3 Use of Flagellin as a Vaccine Adjuvant for Infectious Diseases

### 3.1 *Salmonella typhimurium* Flagellin

The effectiveness of a recombinant *Salmonella typhimurium* flagellin (STF) (RecFlic‐FM; MW: 51 kDa) was assessed as an adjuvant to boost the protective effects of a commercial inactivated anti‐rabies vaccine. This recombinant flagellin was added to the inactivated rabies vaccine Rabikan (strain Shchelkovo‐51) to create enhanced vaccine candidates. Male BALB/c mice, at least 3 weeks old, received intraperitoneal vaccinations with Rabikan formulations that either included recombinant flagellin or lacked an adjuvant. The protective efficacy of these vaccine formulations was evaluated using the National Institutes of Health (NIH) potency test, which is the most widely accepted and recognised method for assessing inactivated rabies vaccines. The results indicated that the specific activity of the rabies vaccine administered alongside the recombinant flagellin adjuvant was significantly greater (48.69 IU/mL) compared to the vaccine without the adjuvant (3.75 IU/mL) [88].

The emergence of multidrug‐resistant (MDR) *Pseudomonas aeruginosa* is becoming a significant concern, especially for chronic lung disease patients. Researchers have explored FLAMOD, a recombinant STF (FliC∆174‐400) modified with an extra amino acid at its N‐terminus, as a promising dual therapy in parallel with the antibiotic gentamicin to address *P. aeruginosa* infections in the face of rising antimicrobial resistance. Administering FLAMOD intranasally as a preventive measure before infection led to a notable decrease in bacterial loads within the lungs, likely due to increased neutrophil recruitment and a decrease in pro‐inflammatory cytokines and inflammatory markers. When FLAMOD was used in conjunction with gentamicin, it demonstrated a synergistic effect, further reducing bacterial counts and enhancing survival rates in a murine model compared to using FLAMOD alone as a pre‐treatment [89].

With poultry cholera caused by various *Pasteurella multocida* serotypes, incorporating adjuvants has shown the potential to enhance the immunogenicity of protein‐based antigens for subunit vaccines. Chung et al. [90] explored the adjuvanticity of recombinant STF in a subunit vaccine aimed at *P. multocida* . For this study, recombinant STF and the antigen fPlpE ( *P. multocida* lipoprotein E) were effectively expressed in *Escherichia coli* for the vaccine formulation. Treatment with flagellin boosted the expression of pro‐inflammatory cytokines, including IL‐1β, IL‐6, and IL‐8, in native chicken peripheral blood mononuclear cells (PBMCs). Additionally, vaccination with formulations containing flagellin led to faster antibody production and provided protection against heterologous *P. multocida* strain A1 in mice, as well as the pathogenic strain Chu01 in poultry.

To further assess the adjuvant properties of STF against infectious bursal disease (IBD) in poultry, researchers engineered two chimeric proteins by fusing the N‐terminal fragments of flagellin FliC99 (residues 1–99) and FliC176 (residues 1–176) with a truncated VP2 protein (tVP2, residues 199–356) from the infectious bursal disease virus (IBDV). These fusion proteins, weighing 48 kDa (FliC99‐tVP2) and 55 kDa (FliC176‐tVP2), were administered subcutaneously to 2‐week‐old brown leghorn chickens in two doses, given 2 weeks apart. The study showed that both chimeric antigen‐adjuvant constructs significantly boosted immune responses, as indicated by increased total immunoglobulin (Ig) G titers. Furthermore, cellular immune responses improved, with higher levels of IFN‐γ and IL‐4. Importantly, the antibodies produced were effective in neutralising IBDV, underscoring the potential of flagellin‐based constructs to enhance the protection of IBD [91].

To create a conjugate vaccine against *S. typhimurium* , lipid‐A free lipopolysaccharide (LFPS) was developed as an immunogen, with recombinant STF that served as an adjuvant along with other protein conjugates. Six‐ to eight‐week‐old BALB/c mice were immunised subcutaneously with different LFPS‐protein conjugates. Among the formulations tested, the LFPS‐STF conjugate produced the strongest immune response against lipopolysaccharide (LPS), leading to significant IgG antibody production and an 80% survival rate by day 28 after pathogen exposure. Significantly, this vaccine showed considerable effectiveness in activating TLR5, even at a low dose of 1 ng/mL [92].

In a similar study, Lai et al. [93] assessed soluble recombinant STF as an adjuvant for creating a safe and effective H5HA subunit vaccine against the highly pathogenic avian influenza virus (AIV) H5N1. When administered intranasally, the combination of the H5HA antigen and STF in an oil‐in‐water emulsion significantly boosted H5HA‐specific IgG and IgA levels in serum, bronchoalveolar lavage fluids (BALFs), and nasal washes. STF‐enhanced mucosal immunisation led to increased IgA levels, especially in mucosal tissues, and raised neutralising antibody levels in serum and BALFs. Furthermore, there was a rise in IgG‐ and IgA‐secreting B cells in the spleen and cervical lymph nodes, along with heightened IL‐17A cytokine production in activated T cells, indicating improved B cell and T cell responses.

Further investigations by Chaung et al. [94] compared the monomeric and polymeric variants of STF (mFliC and pFliC, respectively) as immune activators in specific‐pathogen‐free chickens that were immunised either intramuscularly or intranasally with formalin‐inactivated AIV H5N2 vaccines. The results showed that mFliC, when combined with the 64 unmethylated CpG dinucleotide adjuvant, significantly boosted influenza‐specific plasma IgA antibody levels in chickens vaccinated intramuscularly. Mucosal IgA levels were also notably higher in birds that received mFliC alongside the H5N2 antigen compared to the control group. In contrast, pFliC combined with 64CpG greatly enhanced splenocyte proliferation, further demonstrating its adjuvanticity.

Flagellin has also shown potential as an adjuvant for foot and mouth disease virus (FMDV) vaccines. Hajam et al. [95] explored the effects of recombinant STF when co‐administered with inactivated FMDV antigen in a guinea pig model. When compared to FMDV antigen alone, the combination of flagellin and FMDV antigen through intradermal vaccination resulted in earlier and stronger anti‐FMDV neutralising antibody responses. Both IgG1 and IgG2 antibody isotypes were found to be elevated, although the low IgG1/IgG2 ratio indicated a Th1 cell‐dominated immune response. Moreover, guinea pigs that received the flagellin‐adjuvanted vaccine showed a 70% protection rate against a live viral challenge, in contrast to only 40% protection in those vaccinated with FMDV antigen alone.

Native STF SIN22 and recombinant variants (FliCΔ174‐400 and FliCΔ174‐400/89‐96*) showed strong protective effects against *Streptococcus pneumoniae* infection when given before exposure to the pathogen. In BALB/c mice, administering flagellins 12–24 h before a nasal pneumococcal challenge led to 100% survival, while all untreated mice died from the infection. When flagellin was given alongside *S. pneumoniae* , the survival rate dropped to 75%. Similar protective effects were noted in C57BL/6 and outbred NMRI strain mice. In C57BL/6 mice, flagellin delivered 12 h before the challenge provided 80% protection, whereas NMRI animals achieved complete (100%) protection when the protein was administered 6–32 h before infection. Mechanistically, flagellin enhanced neutrophil infiltration into the respiratory tract and increased the expression of pro‐inflammatory cytokines and chemokines, such as IL‐6, TNF‐α, CXCL1, CXCL2, and CCL20, through TLR5‐mediated signalling pathways [96].

The adjuvant properties of STF were further explored in malaria vaccine formulations using the recombinant 19 kDa C‐terminal region of *Plasmodium vivax* merozoite surface protein‐1 (MSP1_19_). STF, whether combined with or genetically fused to MSP1_19_, effectively triggered strong MSP1_19_‐specific IgG responses. However, intranasal immunisation produced lower antibody titers compared to parenteral immunisation. Notably, spleen cells from mice immunised with the genetically fused MSP1_19_‐STF protein showed higher levels of IFN‐γ release than those immunised with MSP1_19_ combined with STF [97] (Table 1).

**TABLE 1:** Preclinical immune responses and therapeutic potential of flagellin in infectious diseases.

Flagellins	Study type	Target pathogen	Vaccination route	Immune response and therapeutic significance	References
*Salmonella typhimurium* flagellin (STF)	In vivo: Mice	Rabies virus	Intraperitoneal	Enhanced protective efficacy by boosting its immune response about 13 fold	[88]
	In vivo: Mice	MDR *Pseudomonas aeruginosa*	Nasal	Synergistic impact with gentamicin including enhanced neutrophils influx into the lungs and increased mice survival rates	[89]
	In vivo: Mice and Chicken	*Pasteurella multocida*	—	Elicited robust production of pro‐inflammatory cytokines (IL‐1β, IL‐8, and IL‐6) as well as accelerated the antibody response	[90]
	In vivo: Chicken	Infectious bursal disease virus (IBDV)	Subcutaneous	Augmented humoral (total IgG titers), cellular, and cytokine immune responses (IL‐4, IFN‐γ) Increased the synthesis of antibodies necessary to effectively neutralise IBDV	[91]
	In vivo: Mice	*S. typhimurium*	Subcutaneous	Induced a robust immunological response and promoted the synthesis of IgG antibodies, resulting in an 80% survival rate in mice	[92]
	In vivo: Mice	Avian influenza Virus (AIV) H5N1	Nasal	Increased the amount of IgG‐ and IgA‐secreting B cells with elevated production of the IL‐17A cytokine by activated T cells	[93]
	In vitro and In vivo: chicken	AIV H5N2	Intramascular, Nasal	Stimulated TLR5 expression with mucosal antibody production	[94]
	In vivo: Guinea pig	Foot and mouth disease virus (FMDV)	Intradermal	Enhanced Th1‐type responses with a 70% viral protection rate	[95]
	In vivo: Mice	*Streptococcus pneumoniae*	Nasal	Upregulated the expression of IL‐6, TNF‐α, CXCL1, CXCL2, and CCL20 as well as provided resistance to acute pneumonia	[96]
	In vivo: Mice	*Plasmodium vivax*	Nasal, Subcutaneous	Elicited humoral and cellular immune responses with the stimulation of MSP1_19_‐specific serum antibody	[97]
STF phase 2 (STF2)	In vitro and In vivo: Mice	Influenza virus	Nasal, Subcutaneous	Induced strong innate immune response (higher TNF‐α expression) and humoral immunity (M2e‐specific antibody responses)	[98]
	In vivo: Mice	Influenza virus H5N1	Subcutaneous	Induced protective HAI titers that save mice from illness and mortality in a lethal challenge situation	[99]
	In vivo: Mice	Influenza virus H1N1	Subcutaneous	Elicited robust, long‐lasting neutralising antibodies	[100]
	In vivo: Mice and Monkeys	Dengue virus	Subcutaneous, Intramuscular	Generated strong and durable neutralising antibodies (higher GMT)	[101]
Modified version of STF 2 (STF2Δ)	In vivo: Mice	West Nile virus	Subcutaneous	Triggered a robust E‐specific serum IgG response	[102]
*Lactobacillus acidophilus* expressed STF	In vivo: Mice	HIV‐1	Oral	Activated Gag specific cells to secrete IgA	[103]
STF and STFΔ, separately	In vivo: Guinea pig	HIV‐1	Intramuscular, Nasal	Produced higher serum IgG, vaginal IgG and IgA against native Env protein	[104]
*Salmonella enterica* serotype Typhi flagellin	In vivo: Mice	*Salmonella Typhi*	Subcutaneous	Protected mice with appropriate activation of humoral immunity	[105]
	In vivo: Mice	*Yersinia pestis*	Subcutaneous	Elevated the expression of IFN‐γ and TNF‐α with considerable IgG production	[106]
*Salmonella enterica* serovar Enteritidis flagellin	In vitro *and* In vivo: Mice and Monkeys	*Y. pestis*	Intramuscular	Induced TNF‐α expression in vitro Developed strong antigen‐specific humoral immunity	[107]
*Salmonella* flagellin (FliCd)	In vivo: Mice	*Plasmodium yoelii*	Nasal, Subcutaneous	Stimulated CD8^+^ T cell populations that elicited adaptive immunological responses	[108]
*P. aeruginosa* flagellin	In vivo: Mice	HIV‐1	Intradermal	Elicited CTL activity with humoral immune responses	[109]
	In vivo: Mice	*P. aeruginosa*	Subcutaneous	Induced robust cellular and humoral immune responses, including enhanced opsono‐phagocytic killing and pathogen immobilisation at the injury site	[110]
*E. coli* flagellin	In vitro and In vivo: Mice	*E. coli*	Subcutaneous	Enhanced innate and adaptive immune responses by producing TNF‐α, IFN‐γ, and IL‐4 cytokines, as well as anti‐FaeG IgG responses	[111]
*Yersinia ruckeri* flagellin (YRF)	In vitro and In vivo: Channel catfish	—	Intraperitoneal	Elevated expression of IL1‐β1, TNF‐α, IL‐8, iNOS1, and hepcidin after rFlaA, rFlaB, and rFlaC treatment in vitro whereas rFlaC stimulated TLR5M, MHC II, NF‐κB, IL‐8, Hepcidin, TLR5S, IL1‐β1, TNF‐α and iNOS1 in vivo	[112]
	In vitro	—	—	Enhanced the expression of pro‐inflammatory genes including IL‐1β1, IL‐6, IL‐8, and TNF‐α, as well as several acute phase proteins, antimicrobial peptides, and members of the IL‐12 cytokine family	[113]
	In vivo: Rainbow trout	—	Intraperitoneal	Prompt activation of essential pro‐inflammatory cytokines (IL‐1β, TNF‐α, IL‐6, and IL‐11), and chemokines (CXCL‐4 and CXCL‐8) occurred within 6 h, diminishing rapidly by 24 h across several tissues, with a significant increase in IL‐11, IL‐23P19, IL‐17C1, serum amyloid A, and cathelicidin‐2	[114]
	In vivo: Nile tilapia	*Streptococcus agalactiae*	Intraperitoneal	Improved relative percentage survival (RPS) of 59.37%, 71.87%, and 81.25% for the vaccination adjuvanted with flagellin, IFN‐γ, and both, respectively, in contrast to an RPS of 15.62% for the unadjuvanted control group Induced specific IgM antibodies against *S. agalactiae* in the vaccinated cohorts, with a significant augmentation seen after the booster dose Upregulation of MHC II and IgM gene expression in the head kidney and spleen, along with enhanced the activity of immune response parameters such as catalase, lysozyme, superoxide dismutase, and myeloperoxidase	[115]
*Vibrio anguillarum* flagellin	In vitro	—	—	Elicited a 900‐fold and 6‐fold increase in IL‐1β transcription whereas IL‐8 transcription was elevated to 900‐fold and 3‐fold by rFla and rND1, respectively, in gilthead seabream macrophages Enhanced IL‐8 expression by 40‐fold by rFla, whereas rND1 raised the expression by 3‐fold in macrophages of rainbow trout	[116]
	In vitro and In vivo: Atlantic salmon	*Piscirickettsia salmonis*	Intraperitoneal	Rapid increase of pro‐inflammatory cytokines (IL‐1β, IL‐8, IL‐12β), facilitating the production of genes linked to T‐cell activation (IL‐2, CD4, CD8β) and differentiation (IFN‐γ, IL‐4/13, T‐bet, Eomes, GATA3)	[117]
*Vibrio parahaemolyticus* flagellin	In vitro	—	—	Induced NO production, respiratory burst response, and the expression of inflammatory cytokines, such as TnIL‐1β and TnIFN‐γ2	[118]
*Vibrio harveyi* flagellin	In vivo: Orange‐spotted grouper	*V. harveyi*	Intraperitoneal	Increased RPS of 81.8% and 59.1% in fish inoculated with rFlaB and rFlaC, respectively, four weeks post‐vaccination, and to 76.2% and 42.9%, respectively, after eight weeks of vaccination Markedly increased the expressions of IL‐1β, CD4, CD8α, IgM, IFN‐γ, MHC Iα, MHC IIα, TLR5M, and TLR5S in fish injected with rFlaB and rFlaC Induced a higher level of IgM, alkaline phosphatase, lysozyme, acid phosphatase, and superoxide dismutase activity by rFlaB, relative to rFlaC	[119]
	In vivo: Orange‐spotted grouper and Koi carp	*V. harveyi*	Intraperitoneal	Stimulated the production of inflammatory cytokines (IFN‐γ, IL‐1β, and IL‐8) by wild type and ΔMV‐VhFliA in orange‐spotted grouper, while enhanced the expression of IL‐1β, IL‐6, and IL‐8 by wild type and ΔD0MV‐VhFliA in koi carp	[120]
*Edwardsiella tarda* flagellin	In vivo: Turbot	*E. tarda*	Intraperitoneal	Rose of RPS to 70% in turbot immunised with formalin‐killed cells using FlgD as an adjuvant, and enhanced the expression of several immune response‐associated genes, such as MHC I, IgM, IL‐1β, T cell receptor, and TNF‐α in turbot post‐vaccination	[121]

Abbreviations: CTL, cytotoxic T lymphocyte; FaeG, primary subunit of F4 fimbriae; GMT, geometric mean titre; HAI, hemagglutination inhibition; MDR, multidrug‐resistant; MSP1_19_, 19 kDa C‐terminal fragment of *Plasmodium vivax* merozoite surface protein‐1; NO, nitric oxide; RPS, relative percentage survival; TLR5M, membrane bound TLR5; TLR5S, soluble form of TLR5.

Huleatt et al. [98] described a recombinant protein that consists of four tandem repeats of the Influenza M2e antigen linked to the C‐terminus of the STF phase 2 (STF2), referred to as STF2.4 × M2e (VAX102). Mice that were vaccinated with VAX102 in a standard buffer, without any additional adjuvants, produced significantly stronger M2e‐specific antibody responses compared to those injected with M2e peptide mixed with alum. Remarkably, low doses (0.3 μg/mouse) of VAX102 provided protection against lethal challenges with the influenza A virus, significantly improving survival rates (Table 1).

Following this, phase I/II clinical trials (NCT00921947 and NCT00603811) assessed the safety, tolerability, immunogenicity, and incidence of adverse events of VAX102 in healthy adults. The results showed that VAX102 elicited strong M2e‐specific immune responses when given in a prime‐boost regimen. At lower doses ranging from 0.03 to 1.0 μg, it was tolerated satisfactorily, eliciting predominantly mild local or systemic reactions while generating robust M2e‐specific antibody responses, particularly at the 0.3 and 1.0 μg levels. Upon increasing the dosage to 3.0 μg, mild systemic effects, including tiredness and myalgia, were noted with greater frequency; however, these symptoms were self‐limiting and dissipated within 12–18 h. At the maximum provided dosage of 10 μg, 43% of individuals had significant systemic reactions—such as fatigue, fever, headache, and gastrointestinal disturbances—occurring within 2 h post‐injection and subsiding within 24 h. Although cytokine levels were not explicitly measured, the rapid emergence of symptoms and significant elevation in CRP levels, reaching a maximum of 12.5 mg/dL, clearly indicated a temporary cytokine release response induced by TLR5 activation via the flagellin component. The intensity of these inflammatory events did not correspond with the level of M2e‐specific antibody responses, suggesting that the noted reactogenicity mainly resulted from innate immune activation rather than adaptive immunological response. Interestingly, the booster dose did not have an immediate effect on M2e antibody responses, despite the known immunogenicity of flagellin. However, after the booster, there was a noticeable rise in blood CRP levels, along with increased serum IL‐6 levels in certain individuals [28].

Additionally, another randomised, double‐blind, controlled phase I trial (NCT00921973) indicated that administering VAX102 alongside a trivalent inactivated influenza vaccine further boosted immune stimulation, suggesting potential cross‐protection. After getting the vaccine, there was a dose‐dependent increase in pro‐inflammatory cytokines like IL‐6 and TNF‐α. These cytokines spiked right after vaccination but quickly returned to normal levels within 24 h, showing that the immune system was activated in a controlled way. Notably, the study found no serious side effects or widespread inflammatory responses, indicating that while VAX102 effectively enhanced immune responses through TLR5 activation, it did not cause any harmful systemic inflammation. There were some mild local and systemic reactions, such as pain at the injection site and a slight fever, but these are typical side effects associated with vaccines. The findings regarding VAX102 highlighted its safety, immunogenicity, and the possibility of providing protection across multiple influenza seasons without the need for annual reformulations [44] (Table 2). However, no further updates on its clinical trial have been made public.

**TABLE 2:** Clinical trial outcomes on immunogenicity and safety of flagellin‐adjuvanted vaccines for infectious diseases and cancers.

Flagellins	Study type	Key pathogen/cancer type	Vaccination route	Immune responses and trial outcomes	References
*Salmonella typhimurium* flagellin phase 2 (STF2)	Clinical trial: phase I/II	Influenza virus	Intramuscular	Increased serum antibody titers to fourfold Well‐tolerated (doses of 0.03–1 μg) and safe, but associated with elevated levels of CRP at higher doses (3 and 10 μg)	[28]
	Clinical trial: phase I/II	Influenza virus	Intramuscular	Enhanced HAI responses (increase of approximately 1.5‐fold in the GMT) M2e‐specific antibody was 73% seroconverted	[44]
	Clinical trial: phase I/II	Influenza virus H1N1	Intramuscular	Enhanced HAI antibody responses (more than 4‐fold) after receiving higher doses, well‐tolerated (doses of 0.1, 0.3, 1, and 2 μg, without serious adverse events, Increased CRP levels at doses of 3, 5, and 8 μg	[29]
	Clinical trial: phase I/II	Influenza virus H1N1	Intramuscular	Induced HAI antibody more than tenfold among elderly people with well‐tolerated at all doses such as 0.5, 1, 2, 3, 5 or 8 μg	[30]
	In vivo: Rabbit Clinical trial: phase I	Influenza virus H1N1	Intramuscular	Activated immune response (upregulation of TNF‐α, IL‐6, and IL‐8) in rabbits, elevated HAI titers, and high seroconversion and sero‐protection rates	[46]
	Clinical trial: phase I	Influenza virus H1N1, H3N2, B‐YAM, B‐VIC	Intramuscular	Elicited immune responses (increased HAI titers and GMT) at different doses such as 1, 2, 3, 4, 6 μg Pain and swelling at the injection site, as well as headaches, fatigue, and muscle aches were experienced by some patients	[31]
*Salmonella enterica* serovar Enteritidis flagellin	Clinical trial: phase 1	*Y. pestis*	Intramuscular	Well‐tolerated (doses of 1, 3, 6 or 10 μg) vaccination without notable safety concerns Elicited immune response in PBMCs without expression of serum cytokines	[32]
*Salmonella* flagellin (Mobilan)	Clinical trial: Phase I	Prostate	Intraprostatic injection	Temporary rise in total prostate‐specific antigens, accompanied by increased plasma cytokine concentrations (IL‐6, IL‐8, G‐CSF), higher anti‐502s antibody titers, and a greater degree of lymphoid infiltration Safe and well‐tolerated at all doses (1 × 10^9^, 3 × 10^9^, 1 × 10^10^, 3 × 10^10^, and 1 × 10^11^ viral particles for group 1–5 respectively).	[27]
*S. enterica* serovar Dublin flagellin (Entolimod)	Clinical trial: Phase I	Unspecified adult solid tumour	Subcutaneous	Upregulated the plasma cytokine level with reduced levels of suppressive immune cells	[122]

Abbreviations: CRP, C‐reactive protein; GMT, geometric mean titre; HAI, hemagglutination inhibition; PBMCs, peripheral blood mononuclear cells.

VaxInnate researchers enhanced this approach by combining STF2 with the globular hemagglutinin (HA) head protein, resulting in new influenza vaccine candidates [100]. Song and Zhang [99] first attached the HA protein to the C‐terminus of STF2, which led to strong antibody responses and protection against lethal influenza challenges in mice. This same design was then applied to the seasonal influenza A strain (A/Solomon Islands/03/2006), yielding significant immunogenicity in both mice and rabbits (Table 1). Phase I/II clinical trials (NCT00730457) assessed the candidate STF2‐HA (VAX125) vaccine, including STF2 with the globular head of the HA1 domain of A/Solomon Islands/03/2006. When healthy adults received intramuscular injections, there was a noticeable increase in hemagglutination inhibition (HAI) titers that depended on the dose, highlighting the effectiveness of flagellin as a TLR5 agonist, which boosts humoral immunity. Notably, more than half of the participants receiving doses of ≥ 0.5 μg showed fourfold or greater serum HAI responses. However, VAX125 showed a connection to systemic adverse reactions, especially at higher doses. The vaccine led to a dose‐dependent elevation in pro‐inflammatory cytokines, such as IL‐6 and CRP, indicating robust innate immune activation. This reaction correlated with heightened reactogenicity, with unpleasant symptoms including headache, fever, malaise, and myalgia becoming more prominent at elevated dosages. Significant systemic responses were seen at the maximum dosage, impacting 5 out of 128 subjects [29]. In older adults (≥ 65 years), a 5 μg dose of VAX125 achieved an 80% seroconversion rate and a tenfold increase in HAI titers. CRP levels exhibited a dose‐dependent rise, with those receiving the two highest dosages exhibiting a mean CRP spike of less than fivefold. No severe or life‐threatening side events were recorded among the dosing groups, demonstrating that the vaccine was safe in this population [30].

The claim that influenza vaccines did not work well for older adults was somewhat countered by these findings. Although VAX125, a monovalent prototype, demonstrated good immunogenicity and safety at doses of 1–2 μg, its developers raised concerns about how well flagellin would be tolerated in a multivalent vaccine formulation. To address these concerns, VaxInnate developed VAX128, an influenza H1N1 pandemic vaccine based on A/California/7/2009, using three strategies: (1) fusing the HA1 domain to the C‐terminus of STF2 (VAX128A), (2) replacing the D3 domain of STF2 with HA1 (VAX128B), and (3) fusing HA1 to both ends of VAX128B (VAX128C). Immunogenicity and safety assessments in rabbits and humans (NCT01172054) showed that VAX128B and VAX128C had better tolerance and immunogenicity compared to VAX128A at higher doses. Young adults (18–49 years) achieved seroprotective responses at doses of 2.5 μg or more, while elderly participants needed higher doses for similar outcomes. However, older participants showed a dose‐dependent rise in blood CRP levels, with 4.8% of individuals experiencing at least a fourfold increase in serum IL‐6 levels [46]. Tussey et al. [31] further carried out a thorough assessment of the safety, tolerability, and immunogenicity of VAX2012Q, a quadrivalent influenza vaccine that included the STF2 adjuvant along with four hemagglutinin subunits (H1N1, H3N2, B‐YAM, and B‐VIC). This open‐label, multicenter, dose‐ranging phase I clinical trial (NCT02015494) identified treatment‐emergent adverse events related to the vaccine, such as pain and swelling at the injection site, headaches, fatigue, and muscle aches. Despite these side effects, the vaccine produced strong seroprotective immune responses against all components of the influenza virus. However, the study also noted increased levels of IL‐6 and blood CRP (Table 2).

In addition to influenza, VaxInnate investigated tetravalent dengue vaccines (TDVs) by fusing the EIII domain of the dengue virus (DENV) envelope protein to STF2, creating constructs namely R3, C‐term, and R3.2x, like the VAX128 variants. Immunogenicity studies in mice showed strong, long‐lasting neutralising antibody responses (higher geometric mean titres, GMTs) by the R3.2x format that was measured by a 50% focus reduction neutralisation test (FRNT_50_). However, *Rhesus macaques* produced neutralising antibodies to four DENV serotypes when subcutaneously administered with 10 or 90 μg of TDV at 1–1.5 months intervals that were found in the immunogenicity study. In the efficacy study, non‐human primates (NHPs) were vaccinated with 16 μg or 48 μg of TDV at one‐month intervals through the intramuscular route. NHPs that got the latter doses of TDV produced neutralising antibodies against the four serotypes and had decreased viremia after DENV‐2 exposure [101].

VaxInnate previously created a chimeric protein aimed at the West Nile virus by genetically linking a modified version of STF2 (STF2Δ) to the EIII domain of the envelope protein. When administered subcutaneously in mice, this recombinant protein triggered a strong E‐specific serum IgG response, effectively neutralising viral infectivity and providing complete protection against a lethal viral challenge. In contrast, mice that received EIII alone did not produce significant E‐specific antibodies, leading to a survival rate of only 10%. These findings emphasise the essential role of flagellin as a carrier protein in boosting the immunogenicity of vaccine antigens [102].

As it is recognised that human immunodeficiency virus (HIV) transmission primarily occurs through mucosal surfaces, Kajikawa et al. [103] investigated an oral vaccine to stimulate mucosal immunity. They engineered a recombinant strain of *Lactobacillus acidophilus* to express the Gag protein of HIV‐1, either on its own or in combination with STF. Immunogenicity studies in a mouse model showed that mice orally immunised with the recombinant strain expressing both Gag and STF developed Gag‐specific IgA‐producing cells in the large intestine and female reproductive tract. In contrast, mice vaccinated with the strain expressing only Gag did not show significant IgA production, highlighting STF's adjuvant effect on local IgA secretion. Interestingly, the strain expressing just Gag did induce specific IFN‐γ‐producing cells in the mucosa, indicating enhanced cellular immunity. A modified HIV‐1 gp120‐gp41 Env protein, which includes different transmembrane and cytoplasmic domains from the mouse mammary tumour virus glycoprotein, was incorporated into Gag‐derived HIV‐1 virus‐like particles (VLPs). Full‐length STF or a truncated version of STF (STFΔ) was added to the VLPs as adjuvants using a recombinant baculovirus expression system to enhance the immunogenicity of the VLPs. In studies with guinea pig models, the addition of flagellin to VLPs led to improved systemic antibody responses after both parenteral and intranasal vaccinations. Notably, when VLPs containing STF were administered intranasally, there was a significant increase in vaginal mucosal IgA responses. Chimeric VLPs with full‐length STF were more effective at promoting systemic immune responses, while those with STFΔ were particularly good at inducing mucosal IgA production. Importantly, the inclusion of flagellin in the VLPs improved the quality of the antibody responses, as shown by enhanced viral neutralisation capabilities. These results highlight the potential of flagellin variants in customising immune responses for HIV vaccines [104] (Table 1).

While these findings highlighted the promise of using flagellin‐based approaches to boost the effectiveness of HIV vaccines, there were a few hurdles that have slowed their move into clinical use. One major issue was the potential for excessive immune activation. Since flagellin is a powerful TLR5 agonist, it can trigger intense pro‐inflammatory responses, especially at mucosal surfaces, which raised important questions about safety and how well patients could tolerate repeated doses [45, 123]. Technical hurdles surrounding the reliable integration of flagellin into VLPs were quite tricky. On top of that, ensuring consistent quality and immunogenicity from one batch to the next during large‐scale production added another layer of complexity to the development process [124, 125]. Furthermore, pre‐existing immunity to flagellin, resulting from previous exposure to flagellated bacteria, may undermine vaccination effectiveness or modify immune activation [126].

### 3.2 *Salmonella enterica* Serotype Typhi Flagellin

The rise of drug‐resistant bacterial strains, like *S. typhi* , leading to around 10 million gastrointestinal infections annually, highlights the critical need for effective vaccination strategies to fight infectious diseases. Ghorbani et al. [105] investigated the potential of the *S. typhi* flagellin protein as a recombinant vaccine in BALB/c mice. The recombinant flagellin protein, weighing 53.5 kDa, was produced in *E. coli* BL21 and confirmed through sodium dodecyl sulphate‐polyacrylamide gel electrophoresis (SDS‐PAGE) and Western blot analysis. Mice were immunised intraperitoneally with the recombinant protein along with Freund's adjuvant. After vaccination, the mice were exposed to median lethal doses 50 (LD_50_) of live *S. typhi* . The bacterial loads in the liver and spleen were measured, and humoral immune responses were assessed by checking blood IgG levels using an indirect enzyme‐linked immunosorbent assay. The vaccinated mice showed a strong humoral immune response and significantly lower bacterial counts in their livers and spleens compared to the control group.

The plague, a serious and often deadly infectious disease caused by *Yersinia pestis* , continues to pose a significant threat, especially with the increasing issue of antibiotic resistance. Vaccination presents a promising strategy to reduce the impact of such lethal bacterial infections. However, an ideal vaccine for the plague is still not available. Verma et al. [106] developed a recombinant protein by linking *S. typhi* flagellin with *Y. pestis* outer protein YopE, followed by cloning, expression, and purification. BALB/c mice were vaccinated subcutaneously with this recombinant formulation, and its immunomodulatory and protective effects were evaluated. The YopE‐flagellin combination triggered strong humoral immune responses, as shown by increased IgG levels, and robust cellular immunity, indicated by higher production of IFN‐γ and TNF‐α. This vaccine achieved 83% protection against a 100LD_50_ *Y. pestis* challenge in mice, significantly surpassing the 50% protection rate from YopE alone (Table 1).

### 3.3 *Salmonella enterica* Serovar Enteritidis Flagellin

Mizel et al. [107] created a recombinant protein that combines the hypervariable region of flagellin (FliC) from *Salmonella enteritidis* with two protective antigens, F1 and V proteins, from *Y. pestis* to provide protection against respiratory exposure to *Y. pestis* . This recombinant flagellin‐F1‐V protein was recognised by the TLR5 receptor, which activated the production of pro‐inflammatory cytokines, such as TNF‐α, in vitro through the NF‐κB signalling pathway. A prime‐boost vaccination strategy successfully generated strong anti‐F1 and anti‐V humoral immunity in mice and two NHP species. Additionally, the vaccinated mice showed complete bacterial clearance within 3 days after the challenge (Table 1). However, vaccines based on recombinant flagellin may lead to some adverse effects, including systemic cytokine surges or localised inflammation at the injection site, which could limit their clinical application [22]. These potential concerns require careful assessment during human trials. Building on encouraging preclinical findings, Frey et al. [32] evaluated the immunogenicity, safety, and tolerability of the flagellin‐F1‐V recombinant fusion protein in a Phase I clinical trial (NCT01381744). Healthy volunteers aged 18–45 years received increasing doses of the vaccine in phosphate‐buffered saline through intramuscular injection on Days 0 and 28. No significant adverse effects or reactogenicity events were reported while some mild to moderate local and systemic reactogenicity (such as injection site discomfort, headache, and tiredness) was observed, and most importantly, there was no indication of serum cytokine release syndrome. Additionally, no antigen‐specific IgE responses were identified, indicating a limited risk of allergy or hypersensitive reactions. Immune responses to F1, V antigens, and flagellin in PBMCs increased with higher doses, although the 6 and 10 μg doses elicited similar responses (Table 2).

### 3.4 Other *Salmonella* Flagellin

The use of *Salmonella* flagellin (FliCd) as an adjuvant for a malaria T cell vaccine by linking it with the CS280‐288 epitope from the *Plasmodium yoelii* circumsporozoite protein was evaluated by Braga, Massis [108]. They engineered a 56 kDa recombinant protein and assessed its adjuvant properties by immunising pathogen‐free, 8–12‐week‐old female BALB/c mice through either mucosal or subcutaneous routes with the synthetic flagellin protein. The subcutaneous immunisation with the recombinant flagellin effectively triggered specific CD8^+^ T cell responses in the BALB/c mice. To evaluate its ability to stimulate adaptive immunity, CD11c + DCs were examined for MHC I and MHC II expression, as well as activation markers like CD80, CD86, and CD40. The findings showed a slight increase in CD86 and CD40 expression, suggesting that recombinant flagellin could moderately boost DCs activation and play a role in the adaptive immune responses (Table 1).

### 3.5 *P. aeruginosa* Flagellin

The potential of *P. aeruginosa* flagellin as an adjuvant was assessed by comparing its conjugated and mixed forms with a peptide vaccine based on the HIV‐1 p24–Nef candidate. Mice were immunised with the HIV‐1 p24–Nef peptide combined with flagellin in both formats, and various assays were used to evaluate the immune responses. The findings showed that mice receiving the HIV‐1 p24–Nef peptide along with either conjugated or mixed flagellin had enhanced proliferative responses and a Th1 cytokine profile. Importantly, the conjugated version of the vaccine led to better CTL activity, a Th1 cytokine response, and a humoral response dominated by IgM compared to the mixed formulation [109].

In another study, recombinant flagellins from *P. aeruginosa* were tested as a potential vaccine against various *P. aeruginosa* strains in a burn‐injury mouse model. After initial immunisation and two booster doses, a strong immune response was observed. Analysis of serum cytokines showed increased levels of IL‐12 and decreased IL‐10 in mice immunised with flagellin, which correlated with reduced systemic bacterial spread from the initial infection site to the liver and spleen, as well as improved survival rates. The IgG response was mainly of the Th2 type, indicated by higher IgG1 levels and lower IgG2a levels, both before and after the bacterial challenge [110] (Table 1).

### 3.6 *E. coli* Flagellin

Pang et al. [111] explored the adjuvant properties of three flagellin serotypes derived from *E. coli* by cloning and expressing recombinant proteins FliC_H1_, FliC_H7_, and FliC_H19_, which have molecular weights of 66, 64, and 68 kDa, respectively. These flagellin serotypes showed similar effectiveness in both in vitro TLR5 bioactivity assays and in vivo studies evaluating their adjuvanticity. Female BALB/c mice were immunised subcutaneously with FaeG, the main subunit of F4 fimbriae of *E. coli* , along with one of the three recombinant flagellin proteins. The findings indicated that flagellin successfully triggered T cell‐dependent immunity by increasing pro‐inflammatory cytokines such as IFN‐γ, TNF‐α, and IL‐4, thus enhancing both innate and adaptive immune responses. Moreover, CD8^+^ and CD4^+^ T cells showed increased production of IFN‐γ and TNF‐α, while CD4^+^ T cells had decreased IL‐4 secretion. Additionally, all three flagellin serotypes consistently enhanced anti‐FaeG IgG responses in serum, highlighting their potential as effective vaccine adjuvants (Table 1).

### 3.7 *Yersinia ruckeri* Flagellin

The genome of *Yersinia ruckeri* strain SC09 contains three distinct flagellin genes that encode the proteins FlaA, FlaB, and FlaC. Using this strain, researchers successfully expressed the corresponding full‐length recombinant flagellins—rFlaA, rFlaB, and rFlaC, and subsequently investigated their immunostimulatory effects on head kidney monocytes/macrophages derived from channel catfish in vitro. They also looked at the expression patterns of nine immune‐related genes in primary kidney tissues after administering rFlaC. The results showed that all three recombinant flagellins significantly boosted the expression of several pro‐inflammatory markers, such as IL‐1β1, TNF‐α, IL‐8, iNOS1, and the antimicrobial peptide hepcidin. Additionally, treatment with rFlaA, rFlaB, and rFlaC increased the levels of membrane‐bound TLR5 (TLR5M), soluble form of TLR5 (TLR5S), NF‐κB, and MHC IIb in the cultured monocytes/macrophages. Interestingly, rFlaC led to a more pronounced increase in gene expression compared to rFlaA or rFlaB, highlighting its potential as a powerful immunostimulant or adjuvant for aquaculture applications [112].

Earlier research also sought to clarify the immunological role of flagellin in fish infected with flagellated pathogens. Recombinant *Yersinia ruckeri* flagellin (YRF) was synthesised, and its bioactivity was examined using the RTS‐11 macrophage cell line and primary head kidney cells from rainbow trout ( *Oncorhynchus mykiss* ). YRF was found to upregulate pro‐inflammatory genes, including IL‐1β1, IL‐6, IL‐8, and TNF‐α, along with genes responsible for acute‐phase proteins, antimicrobial peptides, and members of the IL‐12 cytokine family [113]. Building on this foundation, Wangkahart, Secombes and Wang [114] further investigated the in vivo immunostimulatory effects of YRF and suggested it could serve as a vaccine adjuvant in aquaculture. Their research assessed the expression of immune‐related genes in both mucosal tissues, like gills and skin, and systemic organs, such as the spleen and liver, after YRF injection. They noted a temporary but strong inflammatory response, with a quick rise in cytokines (IL‐1β, IL‐6, IL‐11, and TNF‐α) and chemokines (CXCL_F4, and CXCL‐8) within just 6 h of administration, which then tapered off by 24 h. This activation was linked to an uptick in antimicrobial effectors, including acute‐phase proteins, antimicrobial peptides, and complement components, especially in the liver and mucosal tissues. Interestingly, they found that IL‐17A/F1, a cytokine associated with Th17 responses, was elevated in the spleen and liver, while IL‐4/13, which indicates Th2 responses, was mainly expressed in the liver. However, the Th1 cytokine IFN‐γ and the anti‐inflammatory cytokine IL‐10 did not show significant increases. While TLR5S was activated in all the tissues they examined, TLR5M was not seen to respond, hinting at a potential regulatory role for TLR5S in controlling excessive inflammation. Notably, the liver showed a heightened sensitivity to flagellin stimulation, with significant increases in the expression of IL‐11, IL‐17C1, IL‐23P19, cathelicidin‐2, and serum amyloid A.

Further supporting the application of flagellin as an immune modulator, Lakshmi, Nandhakumar [115] carried out a study to assess how YRF and IFN‐γ work together to boost the immune response and protective effectiveness in Nile tilapia ( *Oreochromis niloticus* ) that were challenged with *Streptococcus agalactiae* . In this research, they eliminated the minimal contamination from recombinant YRF produced by Wangkahart, Scott [113], and also produced an IFN‐γ derived from Nile tilapia. The fish were vaccinated through intraperitoneal injection with inactivated *S. agalactiae* vaccine that included either YRF, IFN‐γ, or a combination of both. On day 36 after immunisation, the fish were exposed to a virulent strain of *S. agalactiae* and were monitored for 3 weeks. The vaccine provided significant protection, as shown by relative percentage survival (RPS) values of 59.37%, 71.87%, and 81.25% for the groups that received YRF, IFN‐γ, and the combined adjuvant, respectively, compared to RPS of just 15.62% in the control group without any adjuvants. The vaccinated fish produced specific IgM antibodies against *S. agalactiae* , with a notable rise in antibody levels after booster immunisation, especially in the groups that received the adjuvanted formulations. Moreover, gene expression analyses showed a significant increase in MHC II and IgM transcripts in the spleen and head kidney, corresponding with the heightened antibody response. Innate immune factors, such as catalase, lysozyme, superoxide dismutase, myeloperoxidase, and bactericidal activities, were also significantly boosted in the vaccinated groups compared to the controls (*p* < 0.05), further emphasising the immunostimulatory potential of YRF and IFN‐γ as vaccine adjuvants in tilapia (Table 1).

### 3.8 *Vibrio anguillarum* Flagellin

González‐Stegmaier et al. [116] described the production of two recombinant proteins from *V. anguillarum* : the full‐length flagellin B (rFla) and the amino‐terminal part of the D1 domain (rND1); the latter plays a key role in interacting with TLR5 and driving the immunostimulatory effects of flagellin. To evaluate the biological activity of these proteins, they conducted in vitro tests using head kidney macrophages from gilthead seabream ( *Sparus aurata* L., Perciformes, Sparidae) and rainbow trout ( *O. mykiss* W., Salmoniformes, Salmonidae). After 3 h of stimulation with rFla and rND1, macrophages from both fish species showed a significant increase in the pro‐inflammatory cytokines IL‐1β and TNF‐α, along with the chemokine IL‐8. In the gilthead seabream macrophages, rFla and rND1 stimulation led to about a 900‐fold and 6‐fold rise in IL‐1β transcript levels, respectively, while IL‐8 transcription surged by roughly 900‐fold and 3‐fold compared to unstimulated controls. In the rainbow trout, rFla caused a 40‐fold increase in IL‐8 expression, while rND1 resulted in a 3‐fold upregulation compared to non‐stimulated macrophages. In a follow‐up study, González‐Stegmaier et al. [117] investigated the adjuvant effects of rFla and rND1 both in vitro and in vivo during *Piscirickettsia salmonis* vaccination in Atlantic salmon ( *Salmo salar* ). The introduction of these proteins led to a swift rise in pro‐inflammatory cytokines like IL‐1β, IL‐8, and IL‐12β, and enhanced the expression of genes linked to T cell activation (IL‐2, CD4, CD8β) and differentiation (IFN‐γ, IL‐4/13, T‐bet, Eomes, GATA3), with variations depending on the tissue type and timing (Table 1).

### 3.9 *Vibrio parahaemolyticus* Flagellin

*V. parahaemolyticus* is known to be a significant marine pathogen that can trigger serious inflammatory reactions and even death in teleost fish. This not only leads to considerable economic losses in aquaculture but also poses a serious threat to the sustainability of marine fisheries [127]. In a study conducted by Yu et al. [118], DNA, RNA, and total flagellin were extracted from *V. parahaemolyticus* , while also collecting primary spleen and head kidney cells, including leukocytes, from *Tetraodon nigroviridis* . These immune cells were then exposed to the bacterial components they had prepared. The findings showed that when stimulated with total flagellin, there was a remarkable increase in NO production and respiratory burst activity in both the spleen and head kidney cells. Additionally, total flagellin boosted the gene expression and protein production of the pro‐inflammatory cytokine IL‐1β in leukocytes. The study further indicated that flagellin activated the NF‐κB signalling pathway by enhancing luciferase activity in a reporter assay, which was mediated through TLR5M, highlighting the role of TLR5 in innate immune signalling (Table 1).

### 3.10 *Vibrio harveyi* Flagellin

The immunogenic properties of two flagellin antigens from *V. harveyi* , namely FlaB and FlaC, were explored using comparative analysis by Zhang et al. [119]. They developed subunit vaccines (rFlaB and rFlaC) against *V. harveyi* infections and tested the efficacy of the vaccine in the orange‐spotted grouper ( *Epinephelus coioides* ) as their model organism. Four weeks after vaccination, the fish that received rFlaB and rFlaC showed RPS of 81.8% and 59.1%, respectively. By the eight‐week mark, the protective effect had dipped slightly, with RPS values dropping to 76.2% for rFlaB and 42.9% for rFlaC. Immunological tests revealed that both vaccines triggered strong specific antibody responses and boosted innate immune activity compared to the unvaccinated fish. Interestingly, rFlaB significantly increased the levels of IgM and the activity of several innate immune enzymes, like alkaline phosphatase, lysozyme, acid phosphatase, and superoxide dismutase, more than rFlaC did. Additionally, transcriptomic profiling showed that vaccination with either rFlaB or rFlaC led to a noteworthy upregulation of several key immune‐related genes—IL‐1β, CD4, CD8α, IgM, IFN‐γ, MHC Iα, MHC IIα, TLR5M, and TLR5S. These findings confirmed that both antigens could activate innate, humoral, and cellular immune pathways, with rFlaB demonstrating a stronger immunostimulatory effect.

In a separate study, Giovanni et al. [120] took a closer look at the *V. harveyi* flagellin A gene (VhFliA), which they originally isolated from *E. coioides* . They cloned this gene and then explored the immunological effects of the recombinant VhFliA protein that was expressed in *E. coli* . To identify the specific functional domains that trigger immune activation, the researchers created truncated versions of the protein—ΔMV‐VhFliA and ΔD0MV‐VhFliA by—carefully removing certain structural regions. When they tested these in orange‐spotted grouper, they found that both the wild‐type VhFliA and ΔMV‐VhFliA prompted the expression of pro‐inflammatory cytokines like IFN‐γ, IL‐1β, and IL‐8. On the flip side, ΔD0MV‐VhFliA did not spark a similar inflammatory response, indicating that the deleted domains play a crucial role in immune activation. Additional experiments with koi carp ( *Cyprinus carpio* ) backed up these results. The wild‐type VhFliA protein led to a strong increase in IL‐1β, IL‐6, and IL‐8, but the truncated versions showed varied effects: ΔMV‐VhFliA did not boost IL‐1β and IL‐6 levels, while ΔD0MV‐VhFliA did. All in all, these findings highlighted the promise of VhFliA as a powerful immunostimulatory adjuvant in teleosts and revealed important differences in how flagellin influenced TLR5 signalling across different fish species (Table 1).

### 3.11 *Edwardsiella tarda* Flagellin

The immunoprotective efficacy of formalin‐inactivated *E. tarda* vaccine in turbot ( *Scophthalmus maximus* ) was evaluated by incorporating a low dose of *E. tarda* flagellin (FlgD) as an adjuvant. The findings showed that adding FlgD really boosted the vaccine's effectiveness, with the vaccinated turbot showing RPS of 70%. Moreover, the levels of specific serum antibodies reached their highest point 3 weeks after immunisation. Analysing gene expression revealed that several immune‐related markers, such as MHC I, IgM, IL‐1β, T cell receptor, and TNF‐α, were upregulated, indicating that both humoral and cellular immune responses were triggered. These results suggested that FlgD plays a key role in enhancing the protective immunity provided by the inactivated vaccine in turbot [121] (Table 1).

## 4 Potential of Flagellins in Cancer Immunotherapy

The role of TLRs in tumour growth and regression is quite complex, posing significant challenges for the development of effective cancer vaccines. Current strategies focus on slowing cancer progression through various therapeutic approaches. Among these, TLR‐specific ligands have demonstrated considerable potential in triggering and enhancing strong immune responses in different cancers, underscoring their promise for both preventive and therapeutic uses [128]. Flagellin, as a TLR agonist, has been widely researched for its anticancer properties in numerous studies [14, 15, 129, 130].

### 4.1 *Salmonella* Flagellin

Mobilan is a recombinant, nonreplicating adenovirus vector that expresses *Salmonella* flagellin (variant 502s), and it is marketed under the name M‐VM3; this immunotherapeutic drug has been studied for its potential in treating cancer, particularly prostate cancer, as reported by Mett et al. [26]. Their research showed that Mobilan treatment triggered strong immune responses both in vitro and in vivo, leading to significant inhibition of tumour progression in mouse models. When administered directly into tumours of syngeneic prostate cancers in immunocompetent hosts, Mobilan extended survival following surgical tumour removal, mainly by decreasing tumour metastasis. Additionally, in both cell culture and mouse studies, Mobilan produced a more robust and sustained NF‐κB signalling response in the TME, promoting DC activation, T cell infiltration, and reduced tumour metastasis compared to Entolimod, a pharmacologically optimised recombinant protein derived from *Salmonella* flagellin, which was developed previously (Table 3).

**TABLE 3:** Preclinical evaluation of flagellin's therapeutic applications in cancer treatment.

Flagellins	Study type	Tumour/Cancer type	Vaccination route	Immune response and therapeutic significance	References
*Salmonella* flagellin (Mobilan)	In vitro and in vivo: Mice	Prostate	Subcutaneous	Enhanced NF‐κB signalling Suppressed tumour progression	[26]
*S. enterica* serovar Dublin flagellin (CBLB502)	In vivo: Mice and Monkeys	Radiosensitive tissues in HP and GI	Intramuscular	Improved radioprotective Efficacy and did not induce any apparent indicators of toxicity	[131]
*S. enterica* serovar Dublin flagellin (Entolimod)	In vivo: Mice	Lymphoma	Subcutaneous	Activated NK cells, enhancement of co‐stimulatory compounds and pro‐inflammatory cytokines	[132]
	In vivo: Mice	Liver	Subcutaneous	Elicited anti‐tumour activity by stimulating the maturation, differentiation and activation of NK cells and suppressed hepatic metastasis	[133]
	In vivo: Mice	Liver	Subcutaneous	Efficient systemic monotherapy to elicit protective anticancer T cell immunity	[134]
	In vitro and in vivo: Mice	Hepatocellular carcinoma and colorectal cancer	Subcutaneous	Provided excellent protection against LPS‐ and TNF‐induced toxicity in lung and liver Prevented mouse mortality induced by TNF and D‐galactosamine, without suppressing hepatocellular tumour Did not reduce the antitumor efficacy of TNF in the colorectal cancer model	[135]
*S. enterica serovar* Dublin flagellin (GP532)	In vivo: Mice	Radiation‐induced mucositis	Subcutaneous	Enhanced cytokine biomarkers with long lasting effect on NF‐κβ	[136]
STF	In vivo: Mice	Lung	Intranasal	Elicited a tumour‐specific local and systemic pro‐inflammatory cytokine response	[137]
	In vivo: Mice	Melanoma	Subcutaneous	Augmented T cell effector activity and proliferation Decreased immunosuppressive cells	[138]
	In vivo: Mice	Not specified	Intravenous	Enhanced antitumor activity CD4^+^ and CD8^+^ T cells that could suppress the tumour growth	[139]
	In vivo: Mice	TC‐1 cell related tumour	Subcutaneous	Increased strong cellular immunity with anti‐tumour response	[140]
	In vivo: Mice	Melanoma, Colon and Prostate	Intratumoral	Augmented tumour infiltration by CD8^+^ T lymphocytes, together with elevated levels of IFN‐γ and IL‐12 at the tumour spot	[141]

	In vivo: Mice	Breast	Peritumoral	Elicited a contrasting effect on highly immunogenic tumour with its variant due to the improper balance of IFN‐γ and IL‐4 production	[142]
In vitro and in vivo: Mice	Breast	Peritumoral, Intravenous	Upregulated expression of pro‐inflammatory cytokines to induce significant anticancer activity in vitro Inhibited tumour cell proliferation and increased neutrophil infiltration in vivo	[143]
*S. munchen* flagellin	In vivo: Mice	Breast	Subcutaneous	Diminished the dimensions and proliferation rate of the tumour, accompanied by a decreased incidence of metastasis Extended lifespan of vaccinated mice	[144]
*Vibrio vulnificus* flagellin (FlaB)	In vivo: Mice	Cervical	Intravaginal	Induction of CD4^+^ and CD8^+^ T cell recruitment, together with T cell activation‐related gene expression in draining lymph nodes and systemic antigen‐specific IFN‐γ production	[145]
	In vivo: Mice	Cervical	Subcutaneous	Enhanced TLR5 expression that resulted antitumor response and prolonged survival rate	[146]
		Lung	Intravenous	Reduced number of metastatic tumour nodules when receiving with E6/E7 peptides	
*S. typhimurium* secreted FlaB	In vivo: Mice	Colorectal	Intravenous	Elevated the antitumor effectiveness of anti‐PD‐1 treatment by upregulating tumour internal PD‐1 and PD‐L1, and caused death in tumour cells	[147]

Abbreviations: GI, gastrointestinal tract; HP, haematopoietic system.

Eremina et al. [27] carried out a placebo‐controlled, prospective, randomised, single‐blind Phase I clinical trial (NCT02654938) to assess the tolerability, safety, pharmacokinetics, and pharmacodynamics of Mobilan in patients with prostate cancer. The study involved escalating doses (1 × 10^9^, 3 × 10^9^, 1 × 10^10^, 3 × 10^10^, and 1 × 10^11^ viral particles for groups 1–5, respectively) and found that Mobilan was well‐tolerated and safe at all levels. From a pharmacokinetic perspective, it was considered acceptable since there was no Mobilan DNA found in the plasma of patients at any point. Moreover, the level of 502s protein was undetectable in all patient's plasma who received Mobilan, regardless of the time after injection. However, it was noted that patients in the two highest‐dose groups showed increased anti‐502s antibody levels, suggesting that their immune systems were responding to the treatment. The treatment led to a temporary rise in prostate‐specific antigen and cytokine levels such as granulocyte colony‐stimulating factor (G‐CSF) and IL‐6, and increased lymphocyte infiltration in prostate tissues (Table 2). This result raised concerns about the potential for a cytokine storm, which could limit the experiment's effectiveness. Another drawback was the absence of an apparent therapeutic effect, as the Gleason scores showed no significant differences between the Mobilan and placebo groups—both groups demonstrated a similar reduction in cell differentiation. It seemed that the treatment's impact on differentiation might require a longer timeframe to become evident, making it challenging to draw precise conclusions within the study's duration. Furthermore, the immunological activation, which showed the most promising link between safety and pharmacodynamic responses in line with Mobilan's mechanism, was only observed in cohort 5 (received 1 × 10^11^ particles). Additionally, a maximum tolerated dosage could not be determined since all doses given were safe with only minor lab abnormalities (classified as Grade 1 or 2) according to the common terminology criteria for adverse events (CTCAE), and patients reported no medical issues or concerns.

Currently, Panacela Labs is conducting a multicenter, double‐blind, randomised, placebo‐controlled Phase Ib clinical trial (NCT02844699) to evaluate further the efficacy and safety of Mobilan in older prostate cancer patients aged 45–75 years. This ongoing trial is expected to show persistent immunogenicity and clinically significant outcomes, including tumour regression, progression‐free survival, and overall survival. The area requires extensive data on durability, possible immunological memory, and combinatorial ways to convert initial promise into practical, real‐world advantages. As of now, the results from this trial have not yet been published.

While the clinical trial results were promising, several hurdles must be overcome before securing regulatory approval. One primary concern was the risk of immune‐related adverse events, especially systemic inflammation that could occur from the overactivation of TLR5, leading to a spike in pro‐inflammatory cytokines. The temporary rises in IL‐6, IL‐8, and G‐CSF seen in clinical trials highlighted the compound's intended immunostimulatory effects. However, if cytokine production becomes excessive or lasts too long, it could result in systemic toxicity, including potentially dangerous cytokine storm‐like syndromes. Other immune‐related side effects might show up as flu‐like symptoms—fever, shivering, and general malaise—often associated with the activation of the innate immune system. In more serious cases, this inflammatory response could lead to issues like increased vascular permeability, low blood pressure, or organ dysfunction, particularly in patients who are already vulnerable. Additionally, repeated doses might boost immunogenicity, resulting in the development of neutralising antibodies, reduced therapeutic effectiveness, and an increased risk of hypersensitivity reactions. The development of adenoviral vector (non‐replicating) based vaccine production should face strict regulatory scrutiny due to their gene delivery mechanism and the associated risks, such as immunogenicity and insertional mutagenesis. As a result, there was a need for solid evidence regarding how the vector spreads in the body, how the immune system reacts, and its long‐term safety. Moreover, during the scaling up process, it should be essential to follow good manufacturing practices to ensure quality and confirm that there is no replication‐competent adenovirus present. These regulatory and manufacturing requirements could lead to higher production costs and might create barriers to making it widely accessible [27, 29, 148, 149, 150, 151].

Various strategic approaches need to be explored in depth to tackle the risks associated with systemic immune activation and boost the effectiveness of flagellin‐based therapies. Targeted delivery methods, like tumour‐specific viral vectors and nanoparticle encapsulation, are designed to localise immune activation within the TME. This will help to minimise systemic exposure and reduce unwanted inflammatory responses. Additionally, using modified flagellin derivatives that still bind to TLR5 but lessen downstream pro‐inflammatory signalling could be a promising way to reduce cytokine‐related toxicity. Furthermore, combining flagellin‐based treatments with immune checkpoint inhibitors, such as anti‐tumour programmed cell death (PD)‐1 antibody and anti‐cytotoxic T lymphocyte antigen‐4 (CTLA‐4) antibodies, might create synergistic immunotherapeutic effects and help overcome resistance in stubborn tumour types. Fine‐tuning adjuvant formulations is also essential, as it requires careful control of immune activation to avoid excessive or prolonged inflammatory reactions. Finally, conducting long‐term safety assessments is vital for understanding the durability of immune responses and identifying any late‐onset side effects, ensuring these therapies can be safely translated into clinical practices [27, 45, 152, 153, 154].

### 4.2 *Salmonella enterica* Serovar Dublin Flagellin

CBLB502, which was later named Entolimod, was designed to reduce the toxicity and immunogenicity linked to the native flagellin of *Salmonella* Dublin. This was accomplished by incorporating the full C‐ and N‐terminal domains of flagellin, connected by a flexible linker, and producing the protein through an *E. coli* expression system. Preclinical studies indicated that Entolimod lessened the cytotoxic effects of irradiation while maintaining its therapeutic antitumor effectiveness. Notably, it did not increase radiation‐induced tumour formation in mouse and rhesus monkey models [131].

Leigh et al. [132] demonstrated that Entolimod triggered a strong antitumor response by directly activating TLR5‐expressing secondary immune cells, which in turn stimulated CTL. Treatment with CBLB502 revealed protective effects in mouse models of T and B cell lymphoma—both of which do not naturally express TLR5. In T cell lymphoma models, Entolimod protected C57BL/6 mice from tumour‐related death by means that depend on NK cells and perforin‐mediated cytotoxicity. Contrarily, in B cell lymphoma models, Entolimod facilitated tumour elimination in BALB/c mice via a CD8^+^ T cell‐dependent mechanism. ImageStream flow cytometry's cellular analysis showed that CD11b+ and CD11c+ cells, not NK or T cells, were the ones directly reacting to Entolimod (Table 3).

A phase I clinical trial (NCT01527136) led by Bakhribah, Dy [122] assessed the effects of Entolimod on patients with unresectable solid tumours. The findings indicated that Entolimod stimulated the production of plasma cytokines, including G‐CSF, IL‐6, IL‐8, and IL‐10, without causing a cytokine storm. It also lowered levels of immunosuppressive cytokines (Table 2). Furthermore, Yang, Brackett [133] showed that Entolimod quickly activated TLR5‐NF‐κB signalling in liver cells, inhibiting the growth of both TLR5‐expressing and non‐expressing liver cancers by mobilising and activating both innate and adaptive immune responses.

Brackett et al. [134] noted that the systemic administration of Entolimod triggered a series of cell‐to‐cell communication events, which led to the recruitment of innate and adaptive immune components to the liver. This mechanism helped to inhibit liver metastases and foster long‐term anticancer immunological memory. Additionally, Entolimod improved the resilience of normal tissues against the damaging effects of TNF and endotoxins like LPS [135]. However, the clinical translation of Entolimod faced several regulatory and practical challenges that made its progress difficult, even though the preclinical data looked promising. A major obstacle was its elevated immunogenicity, as nearly all patients developed neutralising antibodies shortly after just one dose, which restricted its suitability for repeated or long‐term use—a significant drawback for treatments intended for chronic conditions or those requiring multiple doses. The situation was further complicated by pre‐existing antibodies in some patients, necessitating pre‐treatment screening and potentially narrowing the pool of eligible participants. Additionally, while Entolimod had demonstrated the ability to protect normal tissues from various stresses such as radiation and chemotherapy, its direct antitumor efficacy remained limited, making it challenging to define clear clinical outcomes, particularly since its primary benefit lay in supportive care. Safety concerns were also raised due to its potential to activate NF‐κB signalling and induce cytokines like G‐CSF, increasing the risk of systemic immune activation. As a result, regulatory authorities required comprehensive data to ensure that such immune responses were controllable and reversible [135, 136].

To overcome these limitations, Mett et al. [136] introduced GP532, a new TLR5 agonist and deimmunized variant of Entolimod. By eliminating the epitopes that trigger neutralising immunogenicity, GP532 was able to maintain the pro‐inflammatory effects of Entolimod while minimising the neutralising antibody response. Additionally, GP532 showed enhanced bioavailability and pharmacokinetic characteristics, with radioprotective effects comparable to Entolimod's. Notably, after repeated doses, GP532 was not detected by anti‐Entolimod antibodies and did not produce measurable anti‐GP532 antibody levels in mice. However, it is still uncertain whether GP532 is completely deimmunized. Moreover, its potential role as an adjuvant alongside vaccination antigens has not yet been investigated, indicating a need for further research [20] (Table 3). While flagellin‐mediated NLRC4 activation has been shown to augment antigen cross‐presentation and facilitate strong memory CD8^+^ T cell responses, GP532 lacks the NLRC4‐activating domain, indicating a restricted ability to foster long‐term CTL memory. Another concern was the possible diminished adjuvant effectiveness of GP532 in both mucosal and systemic tissues. Previous research suggested that removing structural regions which were not directly associated with PRR interaction undermined PRR activation and in vivo stability. Consequently, GP532 might have been less effective compared to natural flagellin or Entolimod in maintaining functional integrity at the injection site, due to the excision of structure‐stabilising but PRR nonbinding regions [20, 155].

### 4.3 *Salmonella typhimurium* Flagellin

This flagellin has shown considerable promise in anticancer therapies due to its ability to modulate immune responses and decrease tumour burden. STF was conjugated with the MHC II P10 peptide, which is derived from the *Paracoccidioides brasiliensis* gp43 surface protein and produced in an *E. coli* expression system. When these fusion proteins were delivered intranasally in a mouse melanoma model, there was a significant reduction in lung metastases and a marked improvement in survival rates among the mice. This protective effect was linked to the activation of tumour‐specific CD4^+^ T cells and the enhancement of the MyD88‐dependent signalling pathway [137].

Further progress was made by Geng et al. [138], who showed that tumour‐reactive T lymphocytes engineered to release bacterial flagellin provided a co‐stimulatory signal that improved anticancer efficacy. Human T cells were genetically modified to express TLR5L (a modified coding sequence from STF) alongside a melanoma antigen receptor. In a murine model, these modified T cells increased cytokine production, proliferation, and cytolytic activity against melanoma cells. The treatment led to greater T cell infiltration, which was associated with higher levels of CCR1 and CXCR3, a reduction in PD‐1^+^Lag3^+^ T cells and CD11b^+^Gr1^+^ myeloid‐derived suppressor cells, and alterations in the chemokine and cytokine profile of the tumours.

In another study, Garaude et al. [139] modified tumour cells to express recombinant STF, which triggered a specific immune response against tumour antigens in vivo. This response activated both cytotoxic and Th cells, leading to tumour growth inhibition through the engagement of the TLR5 and NLRC4/NAIP5 signalling pathways. In a similar study, Lin et al. [140] developed recombinant fusion proteins by conjugating E7m, an inactivated form of the human papillomavirus (HPV) E7 protein, to both termini of STF. These recombinant fusion proteins produced strong cellular immune responses and improved antitumour immunity in murine cancer models.

Yu et al. [141] showed that an adenovirus expressing a fusion protein of STF and Grp170, the largest chaperone in the endoplasmic reticulum, generated significant antitumor activity against B16 melanoma and distant lung metastases. This method was more effective than using STF or native Grp170 alone. The STF‐Grp170 fusion protein improved the ability of DCs to present tumour antigens to CD8^+^ T cells, resulting in robust local and systemic cytotoxic CD8^+^ T cell responses.

The effects of TLR5 activation by purified STF have also been explored in vivo. Sfondrini et al. [142] found that the impact on tumour progression varied based on the immunogenicity of the tumour. In highly immunogenic tumours, flagellin reduced proliferation, while it had a minimal effect on less immunogenic variants. These variations were linked to the IFN‐γ/IL‐4 ratio and the presence of CD4^+^CD25^+^ regulatory T cells. Notably, a synergistic effect was observed when flagellin was used alongside CpG‐containing oligodeoxynucleotides, which completely halted tumour development.

Furthermore, flagellin has also shown potential in treating breast cancer. TLR5, which is highly expressed in the ductal epithelium of normal breast tissue, is involved in the inflammatory response to infections. Cai et al. [143] showed that STF treatment led to the release of various cytokines and chemokines, such as GRO‐α (Growth regulated oncogene‐α), ENA‐78 (Epithelial neutrophil activating peptide‐78), MIP‐3α (Macrophage inflammatory protein‐3α), and MDC (Macrophage derived chemokine), while also inhibiting tumour progression in a xenograft mouse model of human breast cancer. Additionally, they demonstrated that TLR5 activation by STF suppressed the anchorage‐independent growth of breast cancer cells, a key indicator of tumorigenic transformation, and modulated the production of pro‐inflammatory cytokines, presenting a unique therapeutic target for breast cancer treatment. These studies underscore the versatility and promise of flagellin‐based approaches in cancer immunotherapy, highlighting its role in influencing immune pathways and enhancing antitumor responses across different cancer types (Table 3).

### 4.4 *Salmonella munchen* Flagellin

Nathalie and Ruth [144] found that vaccines that combine flagellin with the tumour‐associated antigen mucin 1 (MUC1) can trigger a robust Th1‐type immune response against MUC1 while avoiding autoimmunity. In their research, they conjugated flagellin from *Salmonella munchen* with MUC1 to create a fusion protein, which they then tested in vivo through both preventive and therapeutic vaccination trials. The results showed a notable decrease in tumour growth in established mouse tumour models. Furthermore, in an animal model that mimicked grade IV breast cancer, these chimeric vaccines successfully hindered the metastatic process (Table 3).

### 4.5 *Vibrio vulnificus* Flagellin

Recombinant *Fibrio vulnificus* flagellin (FlaB) has been studied as a potent mucosal immunomodulator and potential adjuvant for therapeutic vaccination against cervical cancer. When administered intravaginally (IVAG) alongside E6/E7 peptides, FlaB showed significant tumour suppression and increased survival rates in tumour‐bearing mice, while intranasal or subcutaneous vaccination did not achieve similar tumour regression in the same experimental setup. The IVAG vaccination with FlaB and E6/E7 peptides led to the proliferation of CD4^+^ and CD8^+^ T cells and an upregulation of T cell activation‐related genes in the genital lymph nodes (gLNs). This method also triggered antigen‐specific IFN‐γ production in both the spleen and gLNs, along with a marked increase in TLR5 expression in gLN cells following vaccination [145].

In a similar way, Nguyen et al. [146] found that the combination of FlaB with the E6/E7 tumour‐associated peptide in TC‐1 tumour‐bearing mice significantly reduced tumour growth and extended survival. This treatment also facilitated the infiltration of CD8+ and CD4+ T cells, stimulated antigen‐specific IFN‐γ production, and increased TLR5 expression in immune cells.

More recently, Tian et al. [147] explored the anticancer effects of a combination therapy that included an attenuated *S. typhimurium* strain designed to release FlaB along with anti‐PD‐1 antibody. Their findings indicated that FlaB boosted the infiltration of immune cells, such as M1 macrophages, DCs, and CD8^+^ T cells, into the TME. Additionally, FlaB raised PD‐1 and programmed cell death ligand (PD‐L)‐1 expression through the activation of the AKT/PI3K pathway. This combination therapy showed enhanced efficacy in murine models with tumours, suggesting that FlaB could improve the therapeutic effectiveness of anti‐PD‐1 treatments for patients with colorectal cancer (Table 3).

## 5 Future Insights

Future research should prioritise improving molecular design in flagellin derivatives to increase their immunogenicity and reduce potential adverse effects. Recent advancements in technologies such as CRISPR/Cas9 for antigen engineering enable modifications of flagellin structures, potentially enhancing targeted TLR5 activation while minimising pro‐inflammatory signalling [156, 157]. The integration of flagellin with various immunotherapy modalities, including immune checkpoint inhibitors such as anti‐PD‐1, anti‐PD‐L1, and anti‐CTLA‐4 antibodies, has the potential to augment T cell‐mediated immune responses, presenting a promising strategy for addressing cancer and chronic viral infections [152, 154]. Simultaneously, advancements in nanotechnology necessitate the development of biodegradable nanoparticles, liposomes, and VLPs to facilitate the targeted delivery of flagellin to specific tissues. This targeted approach will minimise systemic cytokine release and enhance safety. Moreover, encapsulation technologies enhance the stability of flagellin and facilitate a controlled release of the antigen, thereby improving its adjuvant properties [38, 42, 74, 153, 158].

The utilisation of artificial intelligence (AI) and machine learning is markedly expediting vaccine research. These technologies facilitate the prediction of immunogenic epitopes, the simulation of flagellin‐TLR5 interactions, and the design of enhanced adjuvants with superior immunological profiles [159, 160]. Flagellin, due to its potent immunostimulatory properties, is gaining recognition as a promising candidate for adjuvants, applicable not only to traditional infectious diseases but also to emerging challenges such as multidrug‐resistant bacteria and novel viral threats. Future clinical trials should prioritise the inclusion of a diverse demographic spectrum and a wider range of infectious diseases and malignancies to enhance the generalisability and robustness of flagellin‐based interventions. Additionally, it is essential to assess the duration and efficacy of the immune memory induced by flagellin, especially concerning tumour recurrence and chronic infections. The integration of modern genomic, proteomic, and transcriptomic techniques may yield insights into the influence of flagellin on immune responses, facilitating the development of advanced precision immunotherapies [10, 16, 45, 89, 161].

## 6 Conclusions

Flagellin's versatility as a vaccine adjuvant and carrier protein underscores its potential to improve systemic and mucosal immunity across diverse vaccine platforms. It is recognised for its robust and wide‐ranging immunostimulatory properties, playing a vital role in vaccine development for various infectious diseases and cancers by activating key immunological pathways that enhance both innate and adaptive immune responses. Notably, strategies utilising flagellin, especially STF, have demonstrated impressive immunostimulatory effects against numerous pathogens, including influenza, dengue, West Nile virus, HIV, rabies, and MDR bacteria like *P. aeruginosa* and *S. typhimurium* . Moreover, flagellin‐based immune stimulants show promise in cancer immunotherapy by boosting the immune system's capacity to target several malignancies such as melanoma, lymphoma, lung, colon, cervical, prostate, and breast cancers. A few STF formulations have advanced to clinical trials to assess their tolerability, safety, and efficacy in humans. In parallel, other bacterial flagellins derived from *S. typhi* , *S. enteritidis* , *S*. Dublin, *S*. *munchen, P. aeruginosa, E. coli*, and *V. vulnificus* , are being explored in preclinical studies for their potential in treating a variety of infectious diseases and cancers. These flagellins have been noted to have less toxicity and be more precise in targeting infections and tumour cells. Despite having multiple advantages, no flagellin‐based vaccine has yet been authorised for human use due to challenges such as excessive TLR5 activation, inflammation, and immune‐related adverse effects, especially at high doses or with repeated administration. In addition, pre‐existing immunity to bacterial flagellin could lower its efficacy in certain people. Therefore, comprehensive preclinical and clinical studies are essential to optimise the therapeutic potential of flagellin‐based therapies. These studies must rigorously assess safety, efficacy, and optimal application strategies, encompassing antigen selection, combination approaches, delivery systems, and immune profiling, to address infectious diseases and cancers effectively.

## Conflicts of Interest

The authors declare no conflicts of interest.

## Data Availability

Data sharing not applicable to this article as no datasets were generated or analysed during the current study.
